# Mid51/Fis1 mitochondrial oligomerization complex drives lysosomal untethering and network dynamics

**DOI:** 10.1083/jcb.202206140

**Published:** 2022-08-31

**Authors:** Yvette C. Wong, Soojin Kim, Jasmine Cisneros, Catherine G. Molakal, Pingping Song, Steven J. Lubbe, Dimitri Krainc

**Affiliations:** 1 Department of Neurology, Northwestern University Feinberg School of Medicine, Chicago, IL; 2 Simpson Querrey Center for Neurogenetics, Northwestern University Feinberg School of Medicine, Chicago, IL

## Abstract

Lysosomes are highly dynamic organelles implicated in multiple diseases. Using live super-resolution microscopy, we found that lysosomal tethering events rarely undergo lysosomal fusion, but rather untether over time to reorganize the lysosomal network. Inter-lysosomal untethering events are driven by a mitochondrial Mid51/Fis1 complex that undergoes coupled oligomerization on the outer mitochondrial membrane. Importantly, Fis1 oligomerization mediates TBC1D15 (Rab7-GAP) mitochondrial recruitment to drive inter-lysosomal untethering via Rab7 GTP hydrolysis. Moreover, inhibiting Fis1 oligomerization by either mutant Fis1 or a Mid51 oligomerization mutant potentially associated with Parkinson’s disease prevents lysosomal untethering events, resulting in misregulated lysosomal network dynamics. In contrast, dominant optic atrophy–linked mutant Mid51, which does not inhibit Mid51/Fis1 coupled oligomerization, does not disrupt downstream lysosomal dynamics. As Fis1 conversely also regulates Mid51 oligomerization, our work further highlights an oligomeric Mid51/Fis1 mitochondrial complex that mechanistically couples together both Drp1 and Rab7 GTP hydrolysis machinery at mitochondria–lysosome contact sites. These findings have significant implications for organelle networks in cellular homeostasis and human disease.

## Teaser

Lysosomes are dynamic organelles requiring proper regulation. Wong et al. demonstrate that inter-lysosomal untethering events are driven by mitochondria–lysosome contact sites, which couple together Drp1 and Rab7 GTP hydrolysis machinery via a mitochondrial Mid51/Fis1 oligomerization complex, which is differentially modulated by distinct disease-associated Mid51 mutants.

## Introduction

Elucidating the regulation of lysosomal networks is essential for studying cellular dynamics and pathogenic disease mechanisms (Ballabio and Bonifacino, 2020; Bonam et al., 2019). In particular, the specific machinery involved in reorganizing a highly dynamic lysosomal network in living cells is still not well understood, including the direct role of other organelles in mechanistically driving this pathway. Of note, while lysosomes are known to tether together before lysosomal fusion events, whether lysosomes undergo inter-lysosomal tethering independent of fusion events remains unclear. Furthermore, how lysosomal tethering contributes to the modulation of the overall network is also not known.

The outer mitochondrial membrane protein Mid51 is a key regulator of mitochondrial network dynamics (Palmer et al., 2011; Zhao et al., 2011), but whether it further plays a role in regulating lysosomal dynamics has not been studied. Interestingly, distinct mutations in Mid51 (*MIEF1*) were recently associated with different human diseases. Mutant Mid51(R169W), which is located in the Mid51 oligomerization domain, was found to be a potential candidate genetic variant for Parkinson’s disease (Lubbe et al., 2020 *Preprint*), while Mid51(Y240N), located in its Drp1-binding domain, was linked to dominant optic atrophy (Charif et al., 2021). However, whether and how these distinct Mid51 mutants might differentially regulate lysosomal network dynamics to drive different human diseases is still not known.

Using super-resolution and live microscopy, we demonstrate here that dynamic inter-lysosomal tethers frequently form to modulate lysosomal networks and are distinct from lysosomal fusion events. Inter-lysosomal untethering events are mediated by a coupled Mid51/Fis1 oligomeric complex on the outer mitochondrial membrane, in which Mid51 and Fis1 promote each other’s oligomerization. Fis1 oligomerization recruits the Rab7-GAP (Jofuku et al., 2005; Onoue et al., 2013) to mitochondria to mediate Rab7 GTP hydrolysis at mitochondria–lysosome contact sites, in order to drive inter-lysosomal untethering events and reorganize the lysosomal network. Importantly, this pathway is selectively inhibited by disrupting Fis1 oligomerization: both mutant Fis1, which disrupts Fis1 oligomerization, and oligomerization domain Mid51(R169W) mutant potentially linked to Parkinson’s disease, which also disrupts Fis1 oligomerization, result in inefficient inter-lysosomal untethering events. This ultimately leads to defective lysosomal network dynamics, including disrupted lysosomal motility and lysosomal distribution and misregulated cargo trafficking over time. In contrast, mutant Mid51(Y240N) associated with dominant optic atrophy, which does not disrupt Mid51/Fis1-coupled oligomerization, does not misregulate lysosomal untethering events or downstream lysosomal network dynamics. Finally, as we also demonstrate that Mid51 oligomerization is directly coupled to Fis1 oligomerization, our study further highlights a potential role for mammalian Fis1 in regulating Drp1 GTP hydrolysis through Fis1’s regulation of Mid51 oligomerization. Together, this work identifies a mitochondrial oligomeric Mid51/Fis1 complex that couples Drp1 and Rab7 GTP hydrolysis machinery to control lysosomal untethering and network dynamics, and suggests that misregulation of this pathway may differentially contribute to distinct human diseases.

## Results

### Inter-lysosomal tethering modulates lysosomal network dynamics

**Figure 1.**
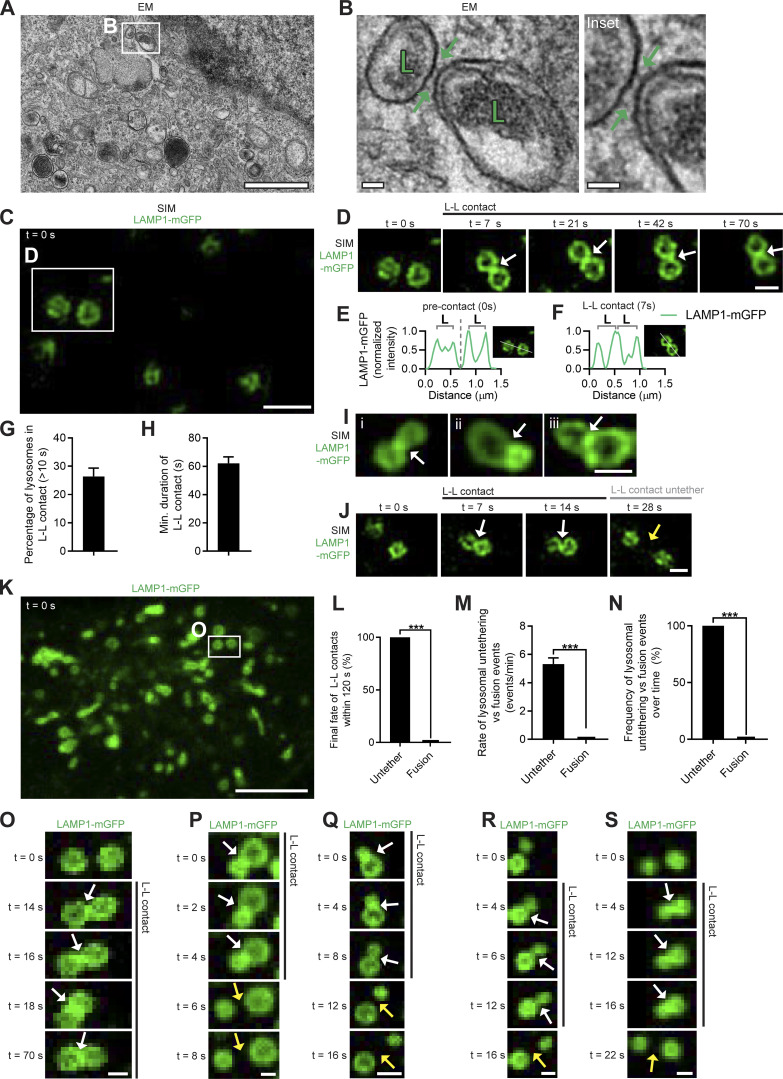
**Inter-lysosomal tethering modulates lysosomal network dynamics. (A and B)** TEM of two lysosomes tethered together (L, arrows) in untreated HeLa cells. Inset (B) shows inter-lysosomal tether. Scale bars, 1 μm (A); 50 nm (B). (**C–F)** Super-resolution SIM of inter-lysosomal (L-L) tethering (white arrows, D) in live HeLa cells (LAMP1-mGFP). Inset (D) shows inter-lysosomal tether formation. Corresponding linescans before tether formation (precontact; *t* = 0 s) and subsequent tethering (contact; *t* = 7 s) are shown in E and F. Scale bars, 1 μm (C); 0.5 μm (D). Video 1 corresponds to D. **(G)** Quantification of percentage of lysosomes in an inter-lysosomal tether (duration >10 s) from confocal live-cell microscopy videos (*n* = 25 cells). **(H)** Quantification of minimum duration of inter-lysosomal tethering (*n* = 88 events from 25 cells). **(I)** Examples of SIM imaging of L-L tethers (white arrows) in live HeLa cells (LAMP1-mGFP). Scale bar, 0.5 μm. **(J)** SIM imaging of L-L tethering (white arrows) and subsequent L-L contact untethering (yellow arrow) in live HeLa cells (LAMP1-mGFP). Scale bar, 0.5 μm. **(K)** Confocal microscopy image of lysosomes in live HeLa cells (LAMP1-mGFP) showing inset corresponding to O (*t* = 0 s). Scale bar, 5 μm. **(L)** The majority of inter-lysosomal tethers undergo untethering events rather than fusion within 120 s of initial contact formation (*n* = 64 events from 25 cells). **(M and N)** Rate of lysosomal untethering events vs. fusion events in live HeLa cells in events/min (M) and frequency of events over time of lysosomal untethering events vs. fusion events (%; N; *n* = 149 total events from 14 cells). **(O–S)** Confocal time-lapse microscopy of L-L tethering (white arrows) and subsequent untethering (yellow arrows) in live HeLa cells (LAMP1-mGFP). Scale bars, 0.5 μm. Video 2 corresponds to S. Mean ± SEM; unpaired two-tailed *t* test (L, M, and N); ***, P < 0.001 (L, M, and N).

**Figure S1.**
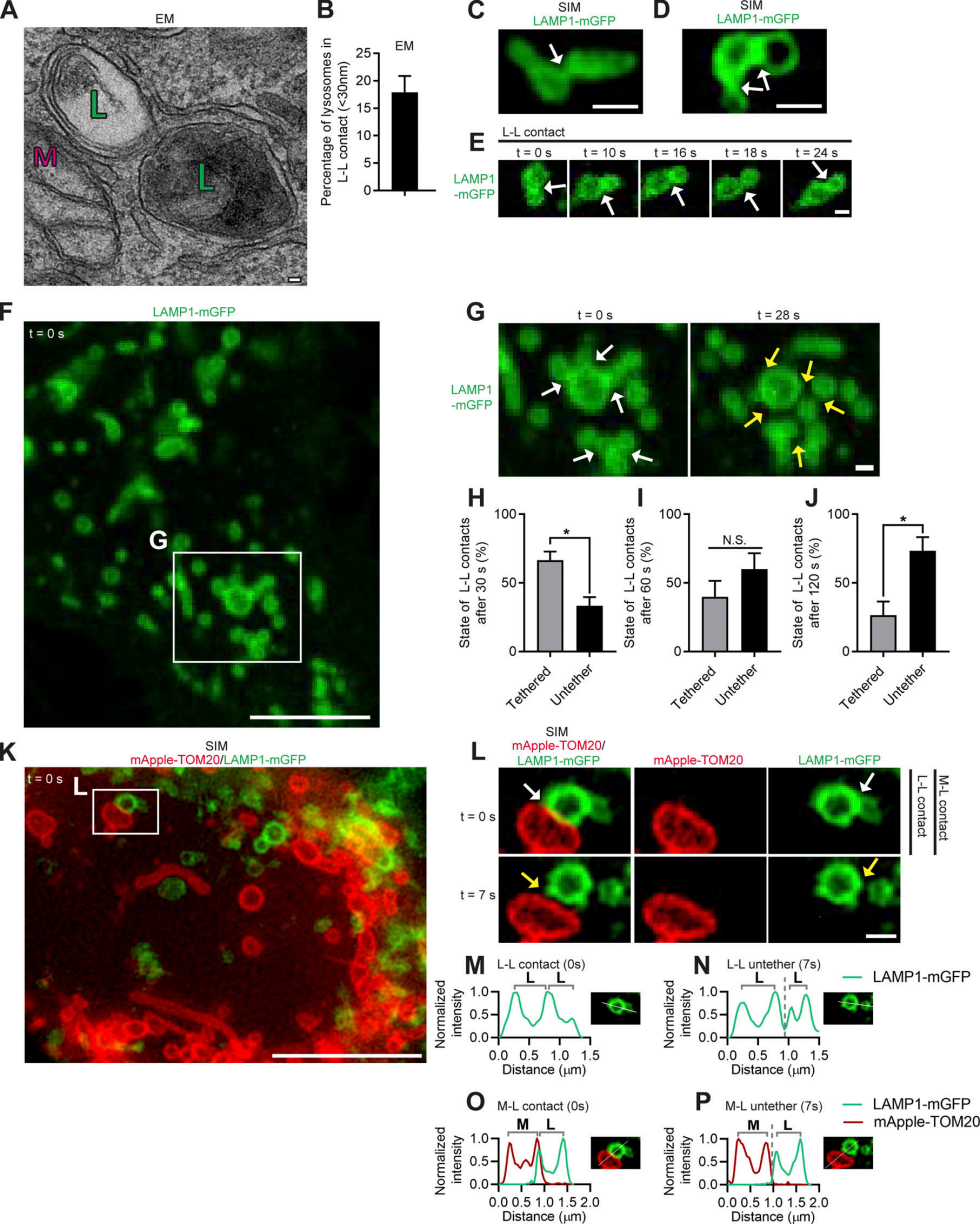
**EM and super-resolution imaging of inter-lysosomal tethering dynamics. (A)** TEM of inter-lysosomal tethering (L) near mitochondria (M) in untreated HeLa cells. Scale bar, 50 nm. **(B)** Quantification of percentage of lysosomes in an inter-lysosomal tether (distance between lysosomal membranes <30 nm) from EM images (*n* = 38 cells). Mean ± SEM. **(C and D)** Super-resolution live-cell SIM of inter-lysosomal tethering (white arrows) in live HeLa cells (LAMP1-mGFP). Scale bars, 0.5 μm. **(E)** Confocal time-lapse imaging of inter-lysosomal (L-L) tethering over time (white arrows) in live HeLa cells (LAMP1-mGFP). Scale bar, 0.5 μm. **(F and G)** Confocal time-lapse microscopy of lysosomal clusters composed of multiple inter-lysosomal tethers (white arrows, inset in G) which subsequently untether (yellow arrows, inset in G). Scale bars, 5 μm (F); 0.5 μm (G). Video 3 corresponds to G. **(H–J)** Quantification of state of inter-lysosomal tether (remain tethered or undergo untethering event) after 30 s (H), 60 s (I) or 120 s (J) of initial tether formation (*n* = 88 events from 25 cells). **(K and L)** Super-resolution live-cell SIM showing L-L untethering event marked by mitochondria–lysosome (M-L) untethering event (yellow arrows, inset in L) in live HeLa cells (mitochondria mApple-TOM20; lysosome LAMP1-mGFP). Scale bars, 5 μm (K); 0.5 μm (L). **(M–P)** Linescans corresponding to L showing inter-lysosomal tethering (L-L) and mitochondria–lysosome tethering (M-L) at *t* = 0 s (M and N) followed by inter-lysosomal untethering and mitochondria–lysosome untethering events at *t* = 7 s (O and P; mitochondria mApple-TOM20; lysosome LAMP1-mGFP). Mean ± SEM; unpaired two-tailed *t* test (H–J); N.S. not significant (I); *, P = 0.02 (H); *, P = 0.029 (J).

From transmission EM (TEM) images, ∼20% of lysosomes were found tethered to another lysosome at an inter-lysosomal tether (<30 nm between lysosomal membranes) in HeLa cells (Fig. 1, A and B; and Fig. S1, A and B; Futter et al., 1996). Inter-lysosomal tethering dynamics were subsequently investigated using live-cell microscopy. By time-lapse super-resolution structured illumination microscopy (SIM) of LAMP1-positive vesicles, inter-lysosomal tethers formed (white arrows; Fig. 1, C and D; and Video 1), and lysosomes were spatially distinct (precontact) before tethering together (L-L contact; Fig. 1, E and F). Consistent with TEM analysis, 26.4 ± 3.0% of lysosomes were in stable inter-lysosomal tethers (duration >10 s; Fig. 1 G), and lysosomes tethered together for 62.2 ± 4.5 s (*n* = 88 examples from 25 cells; Fig. 1 H). Inter-lysosomal tethers frequently formed (Fig. 1 I and Fig. S1, C and D) and subsequently untethered without undergoing fusion (yellow arrows; Fig. 1 J). Importantly, the majority of inter-lysosomal tethers ultimately resulted in untethering events, rather than lysosomal fusion (***, P < 0.001; Fig. 1, K and L). In addition, the rate of lysosomal tethering was significantly higher than the rate of lysosomal fusion, resulting in a greater fraction of untethering events compared with fusion events (***, P < 0.001; Fig. 1, M and N). Inter-lysosomal tether formation (Fig. 1 O), tethering (Fig. S1 E), and untethering events (yellow arrows; Fig. 1, P–S; and Video 2) were further analyzed by confocal time-lapse microscopy in living cells. At the lysosomal network level, multiple lysosomes tethered together (white arrows) to form dynamic clusters that subsequently disassembled over time (yellow arrows) upon multiple untethering events (Fig. S1, F and G; and Video 3). While most inter-lysosomal tethers within the cell persisted after 30 s (Fig. S1 H), the majority had subsequently untethered after 120 s from initial formation (Fig. S1, I and J). Thus, inter-lysosomal untethering events are frequent and temporally regulated events that modulate and reorganize lysosomal networks.

**Video 1.** **Super-resolution microscopy of inter-lysosomal tethering dynamics.** Super-resolution time-lapse SIM of inter-lysosomal tether formation in a live HeLa cell expressing Lamp1-mGFP (lysosome; green). Video was acquired at 1 frame/7 s for 70 s and played back at 3 frames/s (21× speed). Video corresponds to Fig. 1 D. Scale bar, 0.5 µm.

**Video 2.** **Live-cell microscopy of inter-lysosomal untethering event.** Confocal time-lapse microscopy of inter-lysosomal tether formation and subsequent untethering event in a live HeLa cell expressing Lamp1-mGFP (lysosome; green). Video was acquired at 1 frame/2 s for 22 s and played back at 6 frames/s (12× speed). Video corresponds to Fig. 1 S. Scale bar, 0.5 µm.

**Video 3.** **Live-cell microscopy of lysosomal network cluster disassembled by multiple inter-lysosomal untethering events.** Confocal time-lapse microscopy of dynamic lysosomal cluster composed of multiple inter-lysosomal tethers which disassembles over time from multiple untethering events in a live HeLa cell expressing Lamp1-mGFP (lysosome; green). Video was acquired at 1 frame/2 s for 28 s and played back at 3 frames/s (6× speed). Video corresponds to Fig. S1 G. Scale bar, 0.5 µm.

### Mitochondrial contacts promote inter-lysosomal untethering events

**Figure 2.**
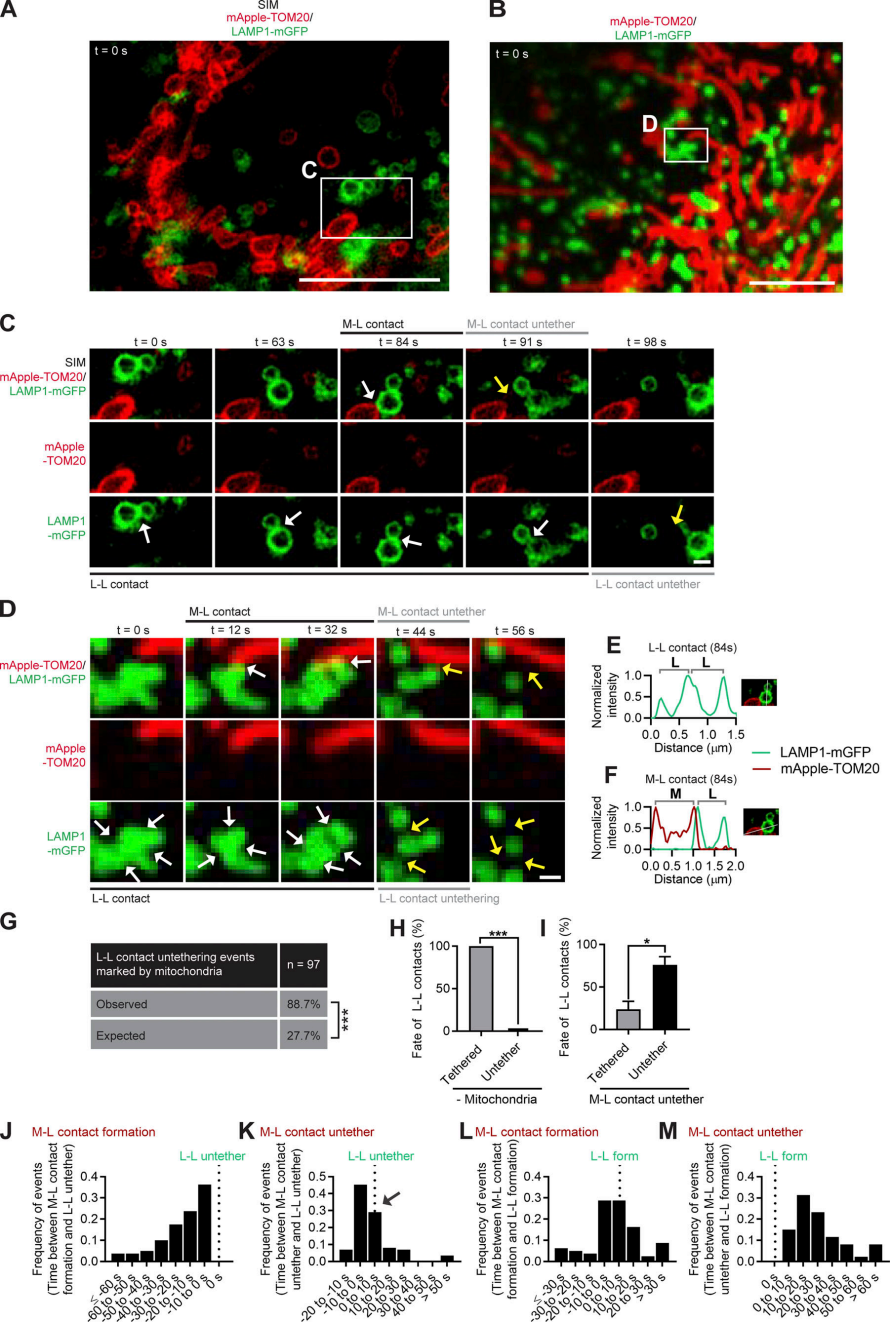
**Mitochondrial contacts promote inter-lysosomal untethering events. (A)** Super-resolution SIM of lysosomes (LAMP1-mGFP) and mitochondria (mApple-TOM20) in live HeLa cells showing inset corresponding to C (*t* = 0 s). Scale bar, 5 μm. **(B)** Confocal microscopy of lysosomes (LAMP1-mGFP) and mitochondria (mApple-TOM20) in live HeLa cells showing inset corresponding to D (*t* = 0 s). Scale bar, 5 μm. **(C)** SIM imaging of inter-lysosomal (L-L) untethering (yellow arrow; bottom) event marked by mitochondria–lysosome (M-L) untethering event (yellow arrow; top) in live HeLa cell (mitochondria mApple-TOM20; lysosome LAMP1-mGFP). Scale bar, 0.5 μm. Video 4 corresponds to C. **(D)** Confocal time-lapse microscopy showing lysosomal cluster of L-L tethers subsequently undergoing untethering events (yellow arrows; bottom) marked by mitochondria–lysosome (M-L) untethering (yellow arrows; top) in live HeLa cells (mitochondria mApple-TOM20; lysosome LAMP1-mGFP). Scale bar, 0.5 μm. Video 5 corresponds to D. **(E and F)** Linescans showing inter-lysosomal tethering (L-L) and mitochondria–lysosome tethering (M-L) from SIM imaging corresponding to C (*t* = 84 s) before untethering events. **(G)** The majority of inter-lysosomal untethering events are marked by mitochondria (Observed), compared with mitochondrial localization by random chance (Expected) in live HeLa cells (*n* = 97 events from 24 cells). **(H and I)** Quantification of fate of L-L tethering (remain tethered or undergo untethering event) after 10 s of no mitochondrial tether (H; *n* = 18 total events from 11 cells (– mitochondria), or a mitochondria–lysosome (M-L) untethering event (I; *n* = 86 total events from 24 cells (M-L contact untether). **(J and K)** Histogram of L-L untethering event compared with mitochondria–lysosome (M-L) formation (J) or untethering (K; *n* >80 events from 24 cells). **(L and M)** Histogram of L-L initial formation compared with mitochondria–lysosome (M-L) formation (L) or untethering (M; *n* >80 events from 24 cells). Mean ± SEM; Fisher’s exact test (G), unpaired two-tailed *t* test (H and I); ***, P < 0.001 (G and H); *, P = 0.0171 (I).

**Figure S2.**
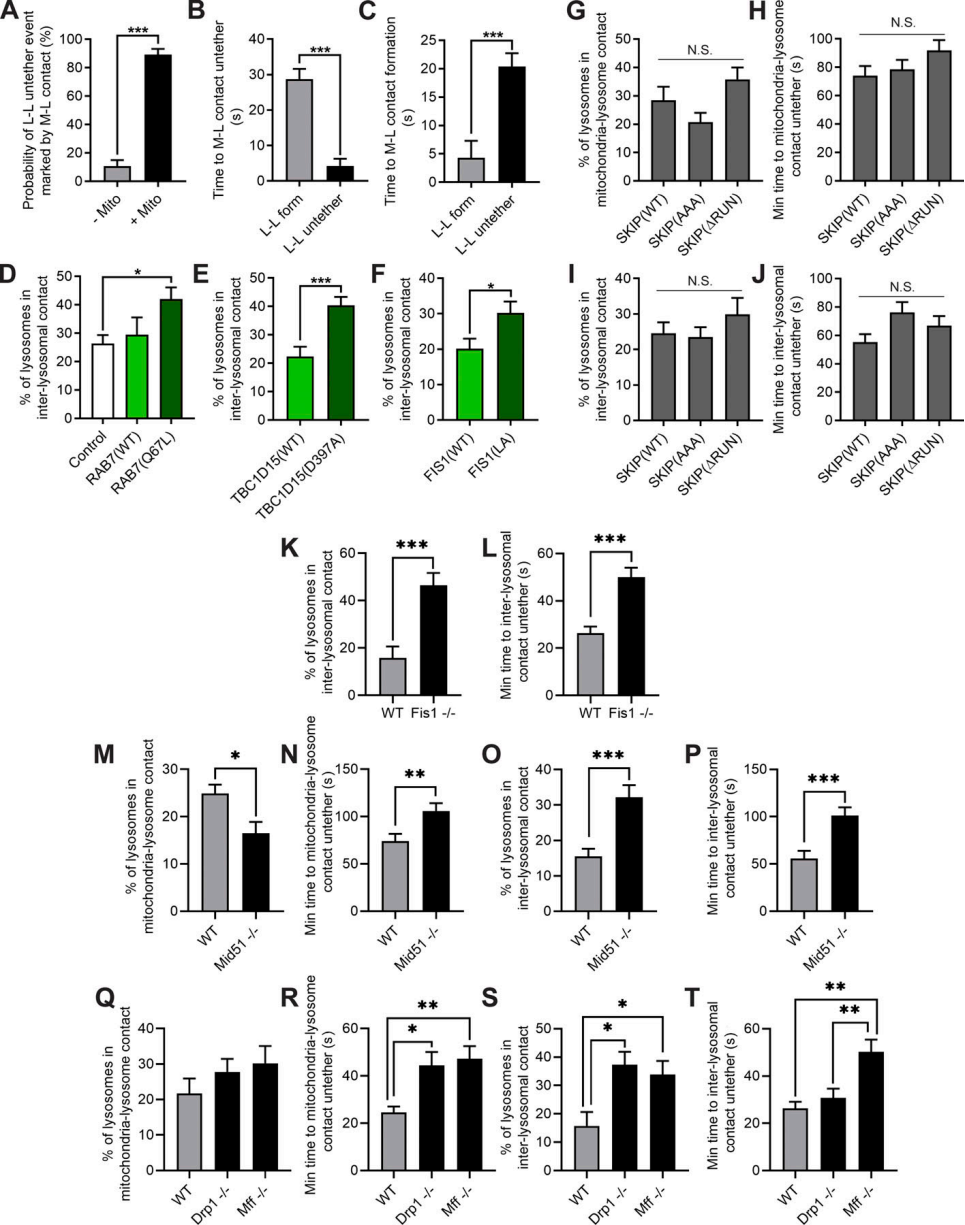
**Rab7 GTP hydrolysis and Drp1 GTP hydrolysis machinery regulate inter-lysosomal tethers. (A)** Percentage of inter-lysosomal (L-L) untethering events marked by no mitochondria (− Mito) or by mitochondria in contact with at least one of the two lysosomes (+ Mito; *n* = 97 total events from 24 cells). **(B)** Mitochondria–lysosome (M-L) untethering is more closely coupled to L-L untethering vs. formation (*n* = 86 total events from 24 cells). **(C)** Mitochondria–lysosome (M-L) formation is more closely coupled to L-L formation vs. untethering (*n* = 80 total events from 24 cells). **(D)** Quantification of increased percentage of lysosomes in inter-lysosomal tether upon inhibition of Rab7 GTP hydrolysis by constitutively active GTP-bound mutant Rab7(Q67L) in live HeLa cells; *n* = 25 cells (Control); *n* = 13 cells (Rab7(WT)); *n* = 15 cells (Rab7(Q67L)). **(E)** Quantification of increased percentage of lysosomes in inter-lysosomal tether by the Rab7-GAP mutant TBC1D15(D397A) that has defective GAP activity in live HeLa cells; *n* = 18 cells (TBC1D15(WT)); *n* = 24 cells (TBC1D15(D397A)). **(F)** Quantification of increased percentage of lysosomes in inter-lysosomal tether by mutant Fis1 (LA) that has defective oligomerization and is unable to recruit TBC1D15 to mitochondria in live HeLa cells; *n* = 26 cells (Fis1(WT)); *n* = 16 cells (Fis1(LA)). **(G and H)** Quantification of percentage of lysosomes in mitochondria–lysosome (M-L) tethers (G) and mitochondria–lysosome (M-L) tethering duration (H) for SKIP(WT) and mutants SKIP(AAA) and SKIP(ΔRUN); *n* = 75 events from 15 cells (SKIP(WT)); *n* = 80 events from 16 cells (SKIP(AAA)); *n* = 75 events from 14 cells (SKIP(ΔRUN)). **(I and J)** Quantification of percentage of lysosomes in L-L tethers (I) and L-L tethering duration (J) for SKIP(WT) and mutants SKIP(AAA) and SKIP(ΔRUN); *n* = 75 events from 15 cells (SKIP(WT)); *n* = 71 events from 16 cells (SKIP(AAA)); *n* = 75 events from 14 cells (SKIP(ΔRUN)). **(K and L)** Loss of Fis1 disrupts inter-lysosomal contact formation (K) and tethering duration (L); *n =* 31 events from 10 cells (WT HCT116); *n =* 75 events from 15 cells (Fis1^−/−^ HCT116). **(M and N)** Loss of Mid51 disrupts mitochondria–lysosome contact formation (M) and tethering duration (N); *n =* 67 events from 15 cells (WT HeLa); *n =* 65 events from 15 cells (Mid51^−/−^ HeLa). **(O and P)** Loss of Mid51 disrupts inter-lysosomal contact formation (O) and tethering duration (P); *n =* 55 events from 15 cells (WT HeLa); *n =* 65 events from 15 cells (Mid51^−/−^ HeLa). **(Q and R)** Loss of Drp1 and Mff disrupt mitochondria–lysosome contact tethering duration (R) but not contact formation (Q); *n =* 47 events from 10 cells (WT HCT116); *n =* 55 events from 11 cells (Drp1^−/−^ HCT116); *n =* 75 events from 15 cells (Mff^−^/^−^ HCT116). **(S and T)** Loss of Drp1 and Mff disrupt inter-lysosomal contact formation (S) and tethering duration (T); *n =* 31 events from 10 cells (WT HCT116); *n =* 55 events from 11 cells (Drp1^−/−^ HCT116); *n =* 72 events from 15 cells (Mff^−^/^−^ HCT116). Mean ± SEM; unpaired two-tailed *t* test (A–C, E, F, and K–P); ANOVA with Tukey’s post hoc test (D, G–J, and Q–T); N.S. not significant (G–J); ***, P < 0.001 (A–C and E); *, P = 0.02 (D); *, P = 0.02 (F); ***, P = 0.0005 (K); ***, P = 0.0006 (L); *, P = 0.0102 (M); **, P = 0.0058 (N); ***, P = 0.0004 (O); ***, P = 0.0003 (P); *, P = 0.031 (R, WT vs. Drp1^−/−^); **, P = 0.0061 (R, WT vs. Mff−/−); *, P = 0.0105 (S, WT vs. Drp1^−/−^); *, P = 0.0305 (S, WT vs. Mff^−/−^); **, P = 0.0054 (T, WT vs. Drp1^−/−^); **, P = 0.0064 (T, WT vs. Mff^−^/^−^).

Next, we examined the temporal regulation of inter-lysosomal untethering. Unexpectedly, mitochondria (TOM20) promoted inter-lysosomal (LAMP1) untethering events as observed by live-cell super-resolution SIM (Fig. 2, A and C; Fig. S1, K and L; and Video 4). Further imaging of lysosomal networks using confocal time-lapse microscopy in living cells revealed that lysosomes tethered in clusters (top, white arrows) were completely disassembled (yellow arrows) after contact with mitochondria (Fig. 2, B and D; and Video 5). Interestingly, before inter-lysosomal contact untethering, lysosomes were in contact both with a lysosome (L-L contact; Figs. 2 E and S1 M) and mitochondria (M-L contact; Figs. 2 F and S1 O), followed by the untethering of both inter-lysosomal and mitochondria–lysosome contacts (Fig. S1, N and P). Mitochondria marked 88.7% of inter-lysosomal untethering events (*n* = 86/97 events from 24 cells), which was significantly greater than expected by chance (27.7%; ***, P < 0.001, Fisher’s exact test; Figs. 2 G and S2 A). Moreover, 100% of inter-lysosomal contacts remained tethered together, if neither lysosome was in contact with mitochondria (Fig. 2 H). In contrast, only 23.8 ± 9.4% of inter-lysosomal contacts remained tethered after mitochondria–lysosome untethering (*n* = 86 events from 24 cells; Fig. 2 I). Further detailed temporal analysis of inter-lysosomal formation/untethering events (Fig. 2, J–M) showed that inter-lysosomal untethering was tightly temporally linked to mitochondria–lysosome untethering events (4.3 ± 2.0 s; Figs. 2 K and S2 B), rather than initial mitochondria–lysosome tether formation (Fig. S2 C). Thus, mitochondria regulate inter-lysosomal untethering and may mechanistically provide the machinery to drive these events.

**Video 4.** **Super-resolution SIM of inter-lysosomal untethering event marked by mitochondria.** Super-resolution time-lapse SIM of inter-lysosomal untethering event marked by mitochondria in a live HeLa cell expressing Lamp1-mGFP (lysosome; green) and mApple-TOM20 (mitochondria; red). Video was acquired at 1 frame/7 s for 105 s and played back at 4 frames/s (28× speed). Video corresponds to Fig. 2 C. Scale bar, 0.5 µm.

**Video 5.** **Live-cell microscopy of lysosomal cluster disassembling after mitochondrial tethering.** Confocal time-lapse microscopy of lysosomal cluster composed of inter-lysosomal tethers which undergo multiple untethering events after contact with mitochondria in a live HeLa cell expressing Lamp1-mGFP (lysosome; green) and mApple-TOM20 (mitochondria; red). Video was acquired at 1 frame/2 s for 56 s and played back at 6 frames/s (12× speed). Video corresponds to Fig. 2 D. Scale bar, 0.5 µm.

### Inter-lysosomal untethering is mediated by Rab7 GTP hydrolysis via mitochondrial TBC1D15/Fis1

**Figure 3.**
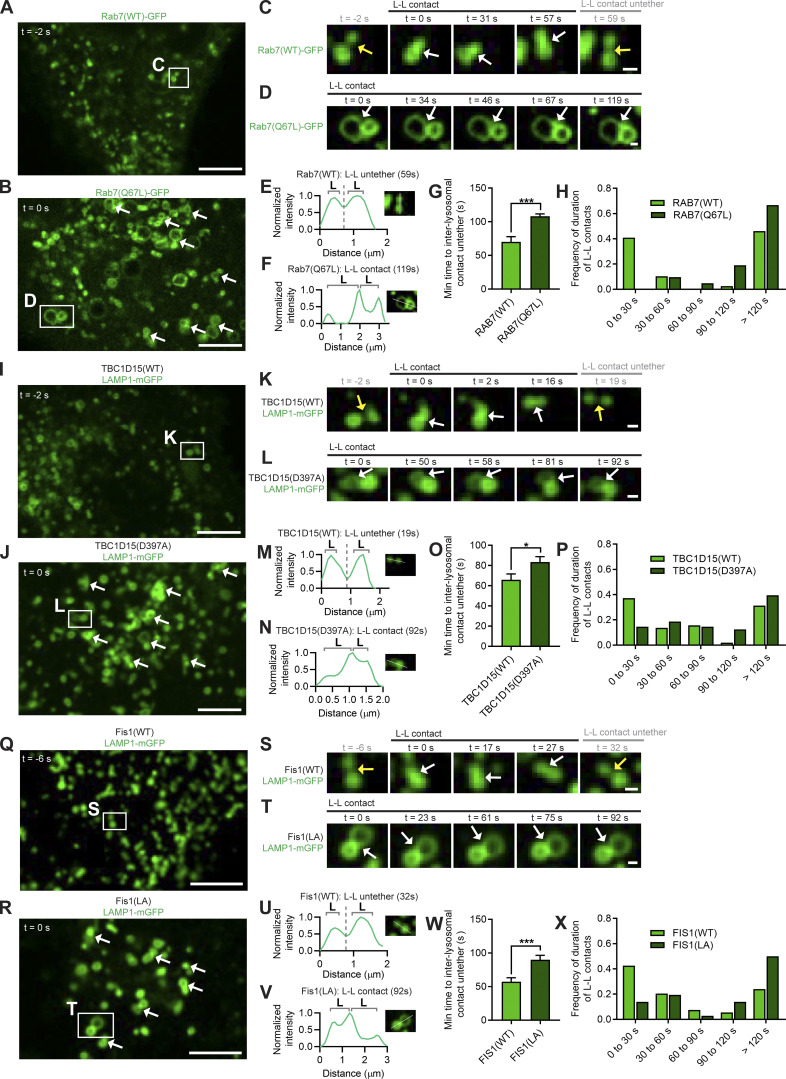
**Inter-lysosomal untethering is mediated by Rab7 GTP hydrolysis via mitochondrial TBC1D15 recruitment by Fis1 oligomers. (A–F)** Confocal time-lapse microscopy showing increased inter-lysosomal (L-L) tether formation (white arrows; A and B) and prolonged tethering (white arrows; insets in C and D) upon inhibition of Rab7 GTP hydrolysis by constitutively active GTP-bound mutant Rab7(Q67L) in live HeLa cells (Rab7(WT)-GFP or Rab7(Q67L)-GFP). Corresponding linescans show L-L untethering for Rab7(WT) (E) vs. prolonged tethering for Rab7(Q67L) (F). Scale bars, 5 μm (A, B); 0.5 μm (C, D). **(G and H)** Quantification and histogram of prolonged L-L tethering duration for Rab7(Q67L) (*n* = 39 events from 13 cells (Rab7(WT)); *n* = 42 events from 14 cells (Rab7(Q67L)). **(I–N)** Confocal time-lapse microscopy showing increased L-L tether formation (white arrows; I and J) and prolonged tethering (white arrows; insets in K and L) by the Rab7-GAP mutant TBC1D15(D397A) that has defective GAP activity in live HeLa cells (lysosome LAMP1-mGFP). Corresponding linescans show L-L untethering for TBC1D15(WT) (M) vs. prolonged tethering for TBC1D15(D397A) (N). Scale bars, 5 μm (I and J); 0.5 μm (K and L). **(O and P)** Quantification and histogram of prolonged L-L tethering duration for TBC1D15(D397A) (*n* = 51 events from 17 cells (TBC1D15(WT)); *n* = 48 events from 16 cells (TBC1D15(D397A)). **(Q–V)** Confocal time-lapse microscopy showing increased L-L tether formation (white arrows; Q and R) and prolonged tethering (white arrows; insets in S and T) by mutant Fis1 (LA) that has defective oligomerization and is unable to recruit TBC1D15 to mitochondria in live HeLa cells (lysosome LAMP1-mGFP). Corresponding linescans show L-L untethering for Fis1(WT) (U) vs. prolonged tethering for Fis1(LA) (V). Scale bars, 5 μm (Q and R); 0.5 μm (S and T). **(W and X)** Quantification and histogram of prolonged L-L tethering duration for Fis1(LA) (*n* = 54 events from 18 cells (Fis1(WT)); *n* = 36 events from 12 cells (Fis1(LA)). Mean ± SEM; unpaired two-tailed *t* test (G, O, and W); ***, P < 0.001 (G and W); *, P = 0.0327 (O).

We then investigated whether inter-lysosomal tethering dynamics might be modulated by the late endosomal/lysosomal regulator Rab7 GTPase (Langemeyer et al., 2018). Indeed, constitutively active GTP-bound Rab7(Q67L). which is unable to undergo GTP hydrolysis, resulted in prolonged inter-lysosomal tethers (Fig. 3, A–F) leading to markedly increased inter-lysosomal tethering duration compared with WT Rab7 (*n* >38 events per condition; ***, P < 0.001; Fig. 3, G and H). Furthermore, Rab7(Q67L) resulted in the increased formation of inter-lysosomal tethers (Fig. S2 D). Interestingly, TBC1D15 is a Rab7 GTPase-activating protein (Rab7-GAP; Zhang et al., 2005; Peralta et al., 2010) that has been shown to be recruited to the outer mitochondrial membrane by mitochondrial protein Fis1 oligomers to drive Rab7 GTP hydrolysis (Jofuku et al., 2005; Onoue et al., 2013; Wong et al., 2018). We found that the GAP mutant TBC1D15 (D397A), which is still recruited to mitochondria but lacks GAP activity (Onoue et al., 2013; Wong et al., 2018), also prolonged inter-lysosomal tethering (Fig. 3, I–N), resulting in increased inter-lysosomal tethering duration (*n* > 48 events per condition; *, P < 0.05; Fig. 3, O and P) and tether formation (Fig. S2 E), suggesting that Rab7 GTP hydrolysis by TBC1D15 drives inter-lysosomal untethering events. Finally, the mitochondrial recruitment of TBC1D15 (Rab7-GAP) requires the oligomerization of the outer mitochondrial membrane protein Fis1 (Jofuku et al., 2005; Onoue et al., 2013). Consistent with our findings that TBC1D15 on mitochondria drives lysosomal untethering, the Fis1(LA) oligomerization mutant, which cannot recruit TBC1D15 to mitochondria (Onoue et al., 2013; Wong et al., 2018), also significantly prolonged inter-lysosomal tethering (Fig. 3, Q–V), leading to increased inter-lysosomal tethering duration (*n* > 35 events per condition; ***, P < 0.001; Fig. 3, W and X) and tether formation (Fig. S2 F). In contrast, mutations in SKIP that disrupted its binding to Rab7 (mutant SKIP(AAA)) or Arl8b (mutant SKIP [ΔRUN]; Jongsma et al., 2020) did not alter the formation or duration of mitochondria–lysosome tethers (Fig. S2, G and H) or inter-lysosomal tethers (Fig. S2, I and J). Thus, while GTP-bound Rab7 promotes lysosomal tethering, TBC1D15, which is recruited to mitochondria via Fis1 oligomerization, drives Rab7 GTP hydrolysis at lysosomal contacts with mitochondria to mediate subsequent inter-lysosomal untethering events and regulate lysosomal network dynamics.

### Coupled oligomerization of a mitochondrial Mid51/Fis1 complex

**Figure 4.**
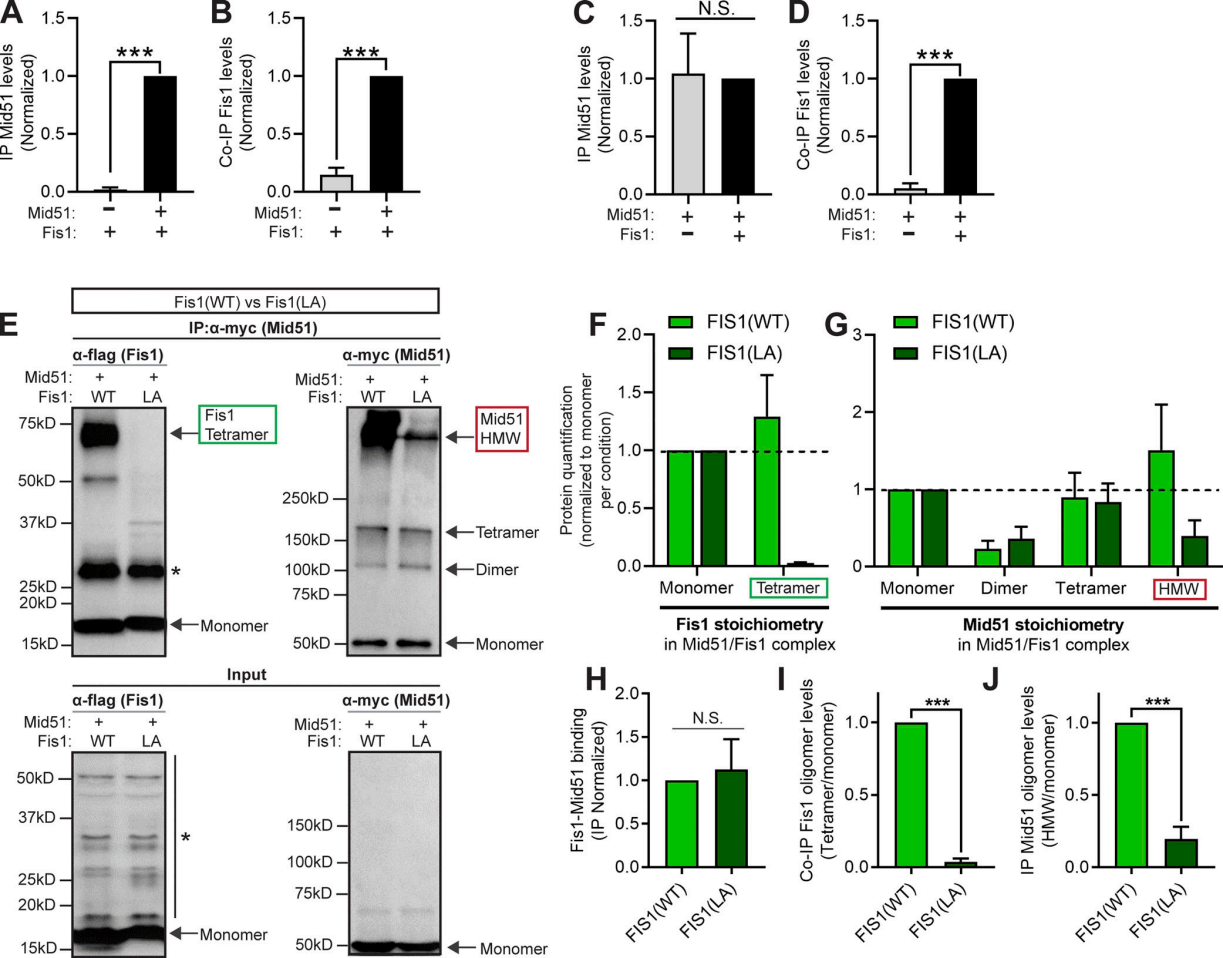
**Coupled oligomerization of a mitochondrial Mid51/Fis1 complex. (A and B)** Quantification of immunoprecipitated WT Mid51 (A) and coimmunoprecipitated WT Fis1 (B) in a Mid51/Fis1 complex, with and without Mid51, confirming that IP of Mid51 and co-IP of Fis1 is dependent on the presence of Mid51 (lane 2); *n* = 3 independent experiments. See Fig. S3 A for representative blots. **(C and D)** Quantification of immunoprecipitated WT Mid51 (C) and coimmunoprecipitated WT Fis1 (D) in a Mid51/Fis1 complex, with and without Fis1, confirming that co-IP of Fis1 is dependent on the presence of Fis1 (lane 2); *n* = 3 independent experiments. See Fig. S3 B for representative blots. **(E)** IP of myc-tagged WT Mid51 and co-IP of Flag-tagged Fis1(WT) or mutant Fis1(LA), with corresponding input. *, Nonspecific bands. **(F and G)** Protein quantification and stoichiometry of the immunoprecipitated mitochondrial Mid51/Fis1 complex with Fis1(WT) or Fis1(LA), revealing Fis1 species (monomer, tetramer; F) and Mid51 species (monomer, dimer, tetramer, and HMW; G), normalized to monomer levels per condition, quantified from IP immunoblot (*n* = 3 independent experiments). **(H–J)** Quantification showing coupled oligomerization of immunoprecipitated Mid51 and coimmunoprecipitated Fis1 in a Mid51/Fis1 complex. Fis1(LA) leads to normal Mid51/Fis1 binding (Fis1 IP monomer/Mid51 IP monomer ratio; H), decreased Fis1 oligomerization (Fis1 IP [tetramer/monomer ratio]; I) and decreased Mid51 oligomerization (Mid51 IP [HMW/monomer ratio]; J); quantified from IP immunoblot; *n* = 3 independent experiments. Mean ± SEM; unpaired two-tailed *t* test (A–D and H–J); ***, P < 0.001 (A, B, D, I, and J); N.S., not significant (C and H). Source data are available for this figure: SourceData F4.

**Figure S3.**
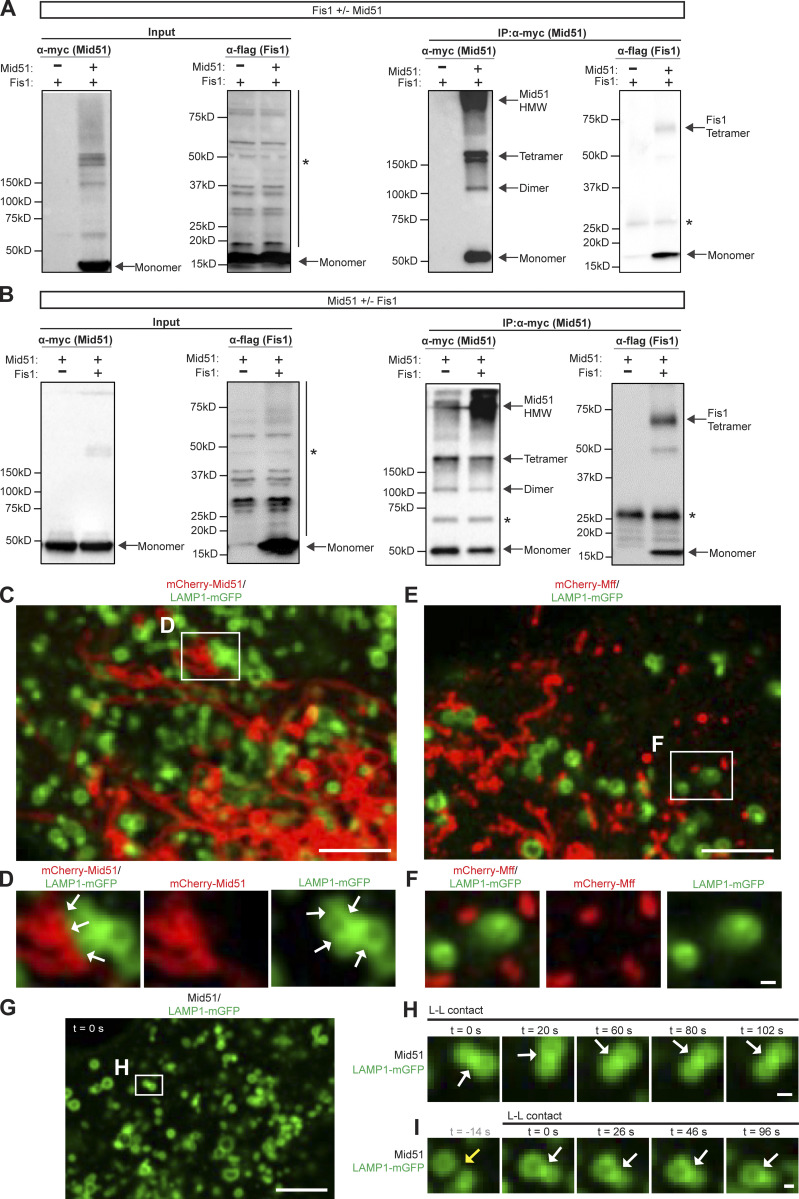
**Mid51 interacts with Fis1 in a Mid51/Fis1 complex and regulates mitochondrial and lysosomal tethering. (A)** IP of myc-tagged WT Mid51 and co-IP of Flag-tagged WT Fis1, with corresponding input, with and without Mid51, confirming that IP of Mid51 and co-IP of Fis1 is dependent on the presence of Mid51 (lane 2). See Fig. 4, A and B, for quantification. *, Nonspecific bands. **(B)** IP of myc-tagged WT Mid51 and co-IP of Flag-tagged WT Fis1, with corresponding input, with and without Fis1, confirming that co-IP of Fis1 is dependent on the presence of Fis1 (lane 2). See Fig. 4, C and D, for quantification. *, Nonspecific bands. **(C and D)** Confocal time-lapse microscopy showing lysosomal cluster of inter-lysosomal tethers which are also tethered to mitochondria (white arrows, inset in D) in live HeLa cells expressing Mid51 (mitochondria mCherry-Mid51; lysosome LAMP1-mGFP). Scale bars, 5 μm (C); 0.5 μm (D). **(E and F)** Confocal time-lapse microscopy showing few inter-lysosomal tethers or mitochondria–lysosome tethers (inset in F) in live HeLa cells expressing Mff (mitochondria mCherry-Mff; lysosome LAMP1-mGFP). Scale bars, 5 μm (E); 0.5 μm (F). **(G–I)**, Confocal time-lapse microscopy showing prolonged inter-lysosomal (L-L) tethering (white arrows; inset in H; I) in live HeLa cells expressing Mid51 (lysosome LAMP1-mGFP). Scale bars, 5 μm (G); 0.5 μm (H and I). Video 8 corresponds to H. Source data are available for this figure: SourceData FS3.

Interestingly, Fis1 binds the outer mitochondrial membrane protein Mid51 (Zhao et al., 2011), which we confirmed by coimmunoprecipitation (co-IP) of WT Fis1 by WT Mid51 (Fig. 4, A–D; and Fig. S3, A and B). Importantly, co-IP of Fis1 by Mid51 was dependent on the presence of both Mid51 (Fig. 4, A and B; and Fig. S3 A) and Fis1 (Fig. 4, C and D; and Fig. S3 B). Next, we investigated whether a Mid51/Fis1 complex on mitochondria might further control lysosomal networks. Mid51 is a mitochondrial adaptor that recruits the mitochondrial fission regulator Drp1 GTPase (Palmer et al., 2011; Zhao et al., 2011). Mid51 oligomerization is known to drive mitochondrial Drp1 oligomerization, which is critical for mitochondrial fission (Koirala et al., 2013; Loson et al., 2014; Kalia et al., 2018), but the role of Fis1 in regulating this pathway in mammalian cells has been unclear. We thus examined whether Fis1 oligomerization was able to regulate the oligomerization of Mid51 (Fig. 4 E). Protein quantification of the coimmunoprecipitated WT Mid51/WT Fis1 complex showed that Fis1(WT) could form monomer and tetramer species (Fis1 co-IP; Fig. 4 F), and Mid51(WT) could form monomer, dimer, tetramer, and high molecular weight (HMW) species (Mid51 IP; Fig. 4 G) in a Mid51/Fis1 complex. Of note, WT Fis1 expression further increased the oligomerization of Mid51 HMW species (Mid51 IP; Fig. S3 B). Thus, WT Fis1 binds Mid51 in a Mid51/Fis1 oligomerization complex on the outer mitochondrial membrane.

We next examined whether the oligomerization mutant Fis1(LA) could still bind Mid51. Mutant Fis1(LA) was still able to coimmunoprecipitate with Mid51 compared with Fis1(WT) (Fig. 4, E and H) and exhibited significantly decreased Fis1 oligomers as expected (Jofuku et al., 2005; Fis1 co-IP [tetramer/monomer ratio]; ***, P < 0.001; Fig. 4, E and I). However, Fis1(LA) also led surprisingly to a striking decrease in the ability of Mid51 to oligomerize in a Mid51/Fis1 complex (Mid51 IP HMW/monomer ratio; ***, P < 0.001; Fig. 4, E and J). Thus, Fis1 oligomers are able to directly promote Mid51 oligomerization in a mitochondrial Mid51/Fis1-coupled oligomeric complex (Model—Steps 1 and 2; Fig. S8 A).

### Regulation of lysosomal tethering dynamics by Mid51

**Figure 5.**
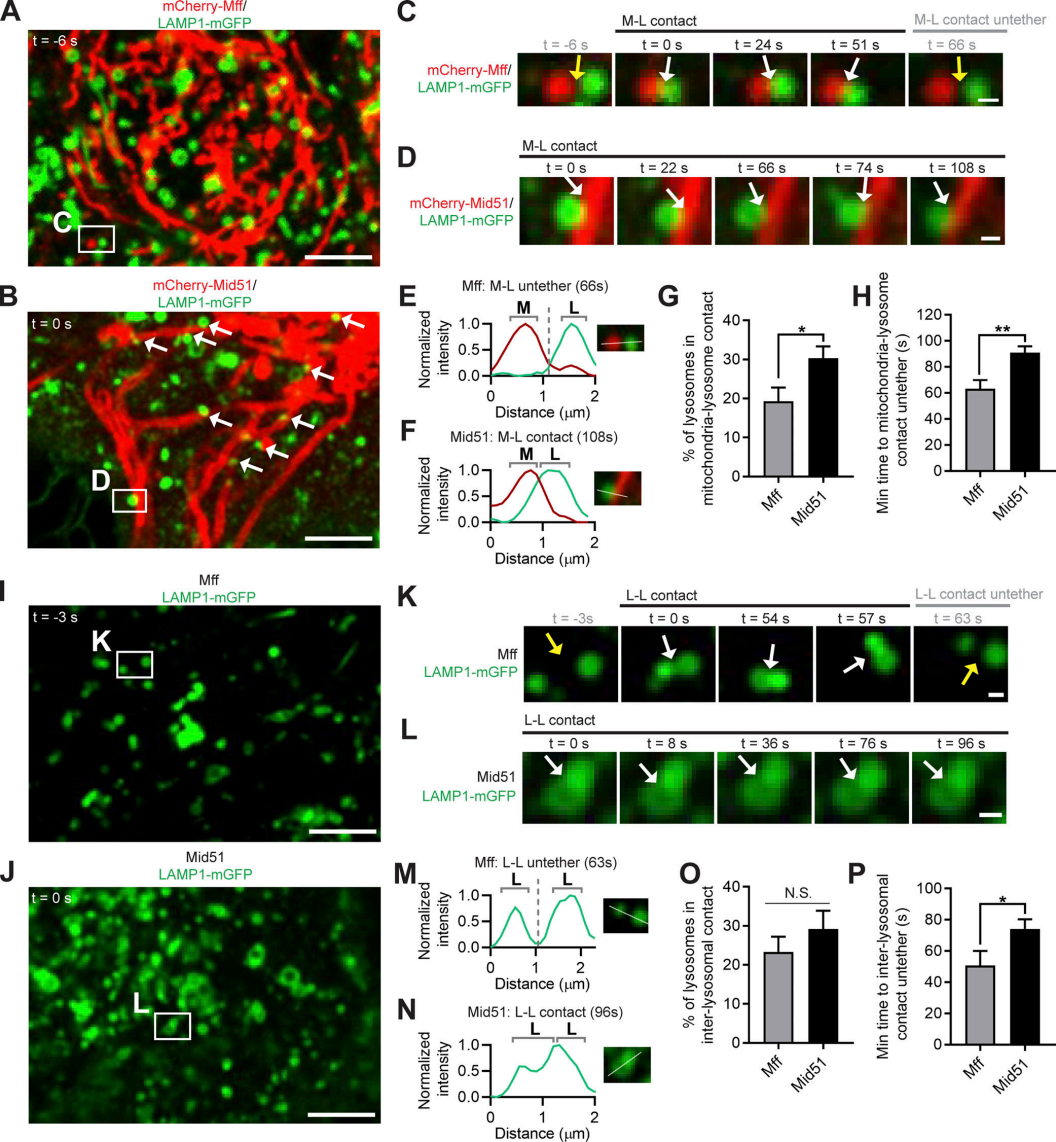
**Regulation of lysosomal tethering dynamics by Mid51 on mitochondria. (A–F)** Confocal time-lapse microscopy showing increased mitochondria-lysosomal (M-L) tether formation (white arrows; A and B) and prolonged M-L tethering (white arrows; insets in C and D) in live HeLa cells expressing Mid51 (mitochondria mCherry-Mid51; lysosome LAMP1-mGFP) compared with Mff (mitochondria mCherry-Mff). Corresponding linescans show M-L untethering for Mff (E) vs. prolonged tethering for Mid51 (F). Scale bars, 5 μm (A and B); 0.5 μm (C and D). Video 6 corresponds to D, and Video 7 corresponds to C. **(G)** Quantification of increased percentage of lysosomes in M-L tethers for Mid51; *n* = 13 cells (Mff); *n* = 25 cells (Mid51). **(H)** Quantification of prolonged M-L tethering duration for Mid51; *n* = 68 events from 13 cells (Mff); *n* = 151 events from 25 cells (Mid51). **(I–N)** Confocal time-lapse microscopy showing lysosomes (I and J) and prolonged inter-lysosomal (L-L) tethering (white arrows; insets in K and L) in live HeLa cells expressing Mid51 (mCherry-Mid51; lysosome LAMP1-mGFP) compared with Mff (mCherry-Mff). Corresponding linescans show L-L untethering for Mff (M) vs. prolonged tethering for Mid51 (N). Scale bars, 5 μm (I and J); 0.5 μm (K and L). **(O)** Quantification of percentage of lysosomes in L-L tethers; *n* = 13 cells (Mff); *n* = 25 cells (Mid51). **(P)** Quantification of prolonged L-L tethering duration for Mid51; *n* = 40 events from 13 cells (Mff); *n* = 68 events from 21 cells (Mid51). Mean ± SEM; unpaired two-tailed *t* test (G, H, O, and P); N.S., not significant (O); *, P = 0.029 (G); **, P = 0.002 (H); *, P = 0.035 (P).

As inter-lysosomal dynamics were regulated by Fis1, we investigated whether inter-lysosomal dynamics might be further modulated by its binding partner Mid51. The adaptor Mid51 inhibits Drp1 GTP hydrolysis when bound (Palmer et al., 2011; Zhao et al., 2011; Osellame et al., 2016), while the adaptor mitochondrial fission factor (Mff) subsequently binds oligomerized Drp1 to drive Drp1 GTP hydrolysis (Gandre-Babbe and van der Bliek, 2008; Otera et al., 2010; Koirala et al., 2013; Liu and Chan, 2015; Clinton et al., 2016; Macdonald et al., 2016; Osellame et al., 2016; Zhang et al., 2016; Yu et al., 2017; Model—Steps 3 and 4; Fig. S8 A). Importantly, Mid51 expression disrupted lysosomal networks, resulting in stably tethered inter-lysosomal clusters (white arrows) that were further tethered to mitochondria, using live imaging of lysosomes (LAMP1) and mitochondria (Mid51; Fig. S3, C and D). In contrast, Mff did not lead to the clustering of lysosomes (LAMP1) or mitochondria (Mff; Fig. S3, E and F). Indeed, mitochondria–lysosome tethers were significantly prolonged over time by Mid51 (Fig. 5, A–F; and Video 6; white arrows in Fig. 5 D) compared with Mff (yellow arrow; untethering in Fig. 5 C and Video 7), and Mid51 significantly increased mitochondria–lysosome tethering formation (Fig. 5 G) and duration (*n* > 65 events per condition; **, P < 0.01; Fig. 5 H). Mid51 also led to inter-lysosomal tethers that were prolonged over time (Fig. 5, I–N; Fig. S3, G–I; and Video 8; white arrows in Fig. 5 L) compared with Mff (yellow arrow; untethering in Fig. 5 K). Further quantitative analysis revealed that Mid51 significantly increased inter-lysosomal tethering duration compared with Mff (*n* >40 events per condition; *, P < 0.05; Fig. 5, O and P), highlighting a role for Mid51 in regulating lysosomal tethering dynamics.

**Video 6.** **Live-cell microscopy of prolonged mitochondria–lysosome tethering by Mid51.** Confocal time-lapse microscopy of prolonged mitochondria–lysosome tethering in Mid51 (WT) condition in a live HeLa cell expressing Lamp1-mGFP (lysosome; green) and mCherry-Mid51 (mitochondria; red). Video was acquired at 1 frame/2 s for 108 s and played back at 12 frames/s (24× speed). Video corresponds to Fig. 5 D. Scale bar, 0.5 µm.

**Video 7.** **Live-cell microscopy of mitochondria–lysosome untethering event by Mff.** Confocal time-lapse microscopy of mitochondria–lysosome formation and subsequent untethering event in Mff condition in a live HeLa cell expressing Lamp1-mGFP (lysosome; green) and mCherry-Mff (mitochondria; red). Video was acquired at 1 frame/3 s for 66 s and played back at 8 frames/s (24× speed). Video corresponds to Fig. 5 C. Scale bar, 0.5 µm.

**Video 8.** **Live-cell microscopy of prolonged inter-lysosomal tethering by Mid51.** Confocal time-lapse microscopy of prolonged inter-lysosomal tethering in Mid51 (WT) condition (mCherry-Mid51) in a live HeLa cell expressing Lamp1-mGFP (lysosome; green). Video was acquired at 1 frame/2 s for 102 s and played back at 12 frames/s (24× speed). Video corresponds to Fig. S3 H. Scale bar, 0.5 µm.

### Drp1 GTP hydrolysis modulates lysosomal tethering dynamics

**Figure 6.**
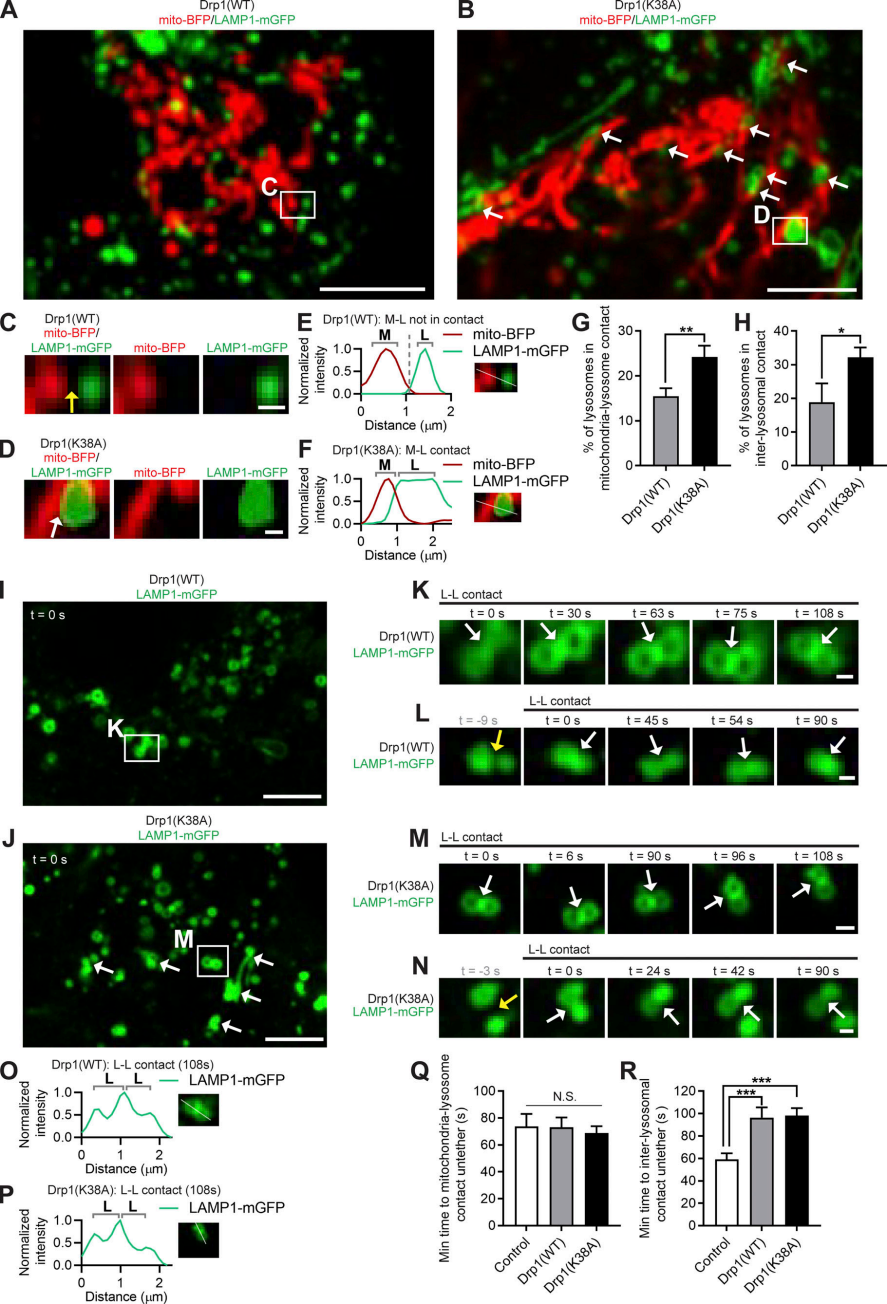
**Drp1 GTP hydrolysis modulates lysosomal tethering dynamics. (A–F)** Confocal time-lapse microscopy showing increased lysosomes in mitochondria–lysosome (M-L) tether formation (white arrows; A and B) and insets (white arrows; C and D) in live HeLa cells upon inhibition of Drp1 GTP hydrolysis by mutant Drp1 (K38A) (mitochondria mito-BFP [pseudocolored red]; lysosome LAMP1-mGFP). Corresponding linescans show M-L not in contact for Drp1(WT) (E) vs. M-L tethering for Drp1(K38A) (F). Scale bars, 5 μm (A and B); 0.5 μm (C and D). **(G)** Quantification of increased percentage of lysosomes in mitochondria–lysosome (M-L) tethers for Drp1(K38A); *n* = 21 cells (Drp1(WT)); *n* = 20 cells (Drp1(K38A)). **(H)** Quantification of increased percentage of lysosomes in inter-lysosomal (L-L) tethers for Drp1(K38A); *n* = 21 cells (Drp1(WT)); *n* = 20 cells (Drp1(K38A)). **(I–P)** Confocal time-lapse microscopy showing increased L-L tether formation (white arrows; I and J) and prolonged L-L tethering (white arrows; insets in K–N) in live HeLa cells expressing Drp1(WT) and Drp1(K38A) (lysosome LAMP1-mGFP). Corresponding linescans show prolonged L-L tethering for Drp1(WT) (O) and Drp1(K38A) (P). Scale bars, 5 μm (I and J); 0.5 μm (K–N). **(Q)** Quantification of mitochondria–lysosome (M-L) tethering duration for Drp1(WT) and Drp1(K38A); *n* = 38 events from 19 cells (control); *n* = 82 events from 21 cells (Drp1(WT)); *n* = 104 events from 19 cells (Drp1(K38A)). **(R)** Quantification of prolonged L-L tethering duration for Drp1(WT) and Drp1(K38A); *n* = 88 events from 25 cells (control); *n* = 36 events from 15 cells (Drp1(WT)); *n* = 58 events from 15 cells (Drp1(K38A)). Mean ± SEM; unpaired two-tailed *t* test (G and H), ANOVA with Tukey’s post hoc test (Q and R); N.S., not significant (Q); **, P = 0.0097 (G); *, P = 0.044 (H); ***, P < 0.001 (R).

As Mid51 inhibits Drp1 GTP hydrolysis when bound (Palmer et al., 2011; Zhao et al., 2011; Osellame et al., 2016), while Mff promotes Drp1 GTP hydrolysis (Gandre-Babbe and van der Bliek, 2008; Otera et al., 2010; Koirala et al., 2013; Liu and Chan, 2015; Clinton et al., 2016; Macdonald et al., 2016; Osellame et al., 2016; Zhang et al., 2016; Yu et al., 2017), we further examined whether Drp1 GTP hydrolysis might thus modulate lysosomal tethering dynamics. Interestingly, the GTP hydrolysis-deficient mutant Drp1(K38A) (Smirnova et al., 1998) increased the percentage of lysosomes tethered to mitochondria (Fig. 6, A–F) compared with Drp1(WT), resulting in a significant increase in the formation of mitochondria–lysosome tethers (**, P < 0.01; Fig. 6 G). Moreover, Drp1(K38A) further increased the formation of inter-lysosomal tethers (*, P < 0.05; white arrows, Fig. 6, H–J). Of note, both Drp1(WT) (white arrows, Fig. 6, K and L; corresponding linescan in Fig. 6 O) and Drp1(K38A) (white arrows, Fig. 6, M and N; corresponding linescan in Fig. 6 P) prolonged inter-lysosomal tethering, leading to increased inter-lysosomal tethering duration (Fig. 6, Q and R). Together, these results highlight a role for mitochondria in regulating tethered lysosomal network dynamics via Drp1 GTP hydrolysis and its mitochondrial adaptor Mid51.

**Figure S4.**
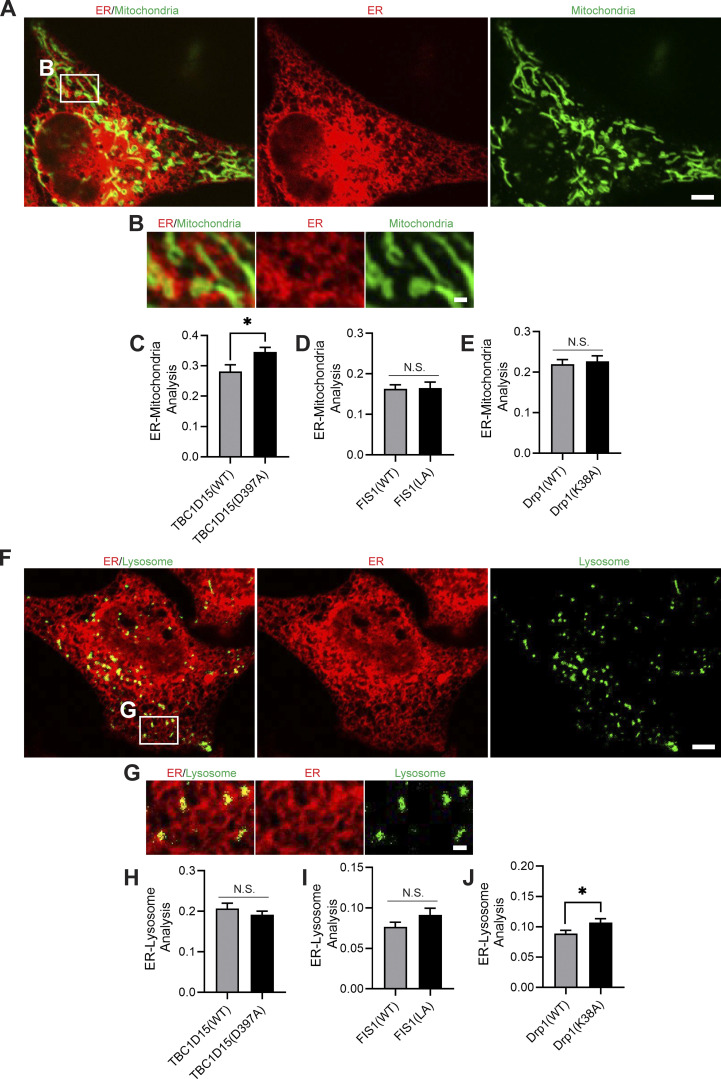
**Regulation of mitochondrial and lysosomal contacts with the ER. (A and B)** Confocal microscopy image of ER contacts with mitochondria (inset in B) in live HeLa cells (ER, red [mCherry-ER]; mitochondria, green [mEmerald-TOMM20]). Scale bars, 5 μm (A); 1 μm (B). **(C–E)** Effect of TBC1D15 (WT vs. D397A), Fis1 (WT vs. LA), and Drp1 (WT vs. K38A) on ER-mitochondria contacts. C, *n* = 45 cells (TBC1D15(WT)); *n* = 57 cells (TBC1D15(D397A)); D, *n* = 33 cells (Fis1(WT)); *n* = 27 cells (Fis1(LA)); E, *n* = 29 cells (Drp1(WT)); *n* = 38 cells (Drp1(K38A)). **(F and G)** Confocal microscopy image of ER contacts with lysosomes (inset in G) in live HeLa cells (ER, red [mCherry-ER]; lysosome, green [mTagBFP2-Lysosomes-20]). Scale bars, 5 μm (F); 1 μm (G). **(H–J)** Effect of TBC1D15 (WT vs. D397A), Fis1 (WT vs. LA), and Drp1 (WT vs. K38A) on ER-lysosome contacts. H, *n* = 45 cells (TBC1D15(WT)); *n* = 57 cells (TBC1D15(D397A)); I, *n* = 33 cells (Fis1(WT)); *n* = 27 cells (Fis1(LA)); J, *n* = 19 cells (Drp1(WT)); *n* = 23 cells (Drp1(K38A)). Mean ± SEM; unpaired two-tailed *t* test (C–E and H–J); N.S., not significant (D, E, H, and I); *, P = 0.0157 (C); *, P = 0.0432 (J).

As both mitochondria and lysosomes can simultaneously form contacts with the ER (Wong et al., 2018), we also examined whether mutations in TBC1D15, Fis1, and Drp1 might further misregulate the formation of these contacts. Live-cell microscopy of mitochondria (green) and ER (red) revealed multiple contacts between the two organelles (Fig. S4, A and B), which were altered by mutant TBC1D15(D397A) (Fig. S4 C) but not by Fis1(LA) or Drp1(K38A) (Fig. S4, D and E). We also investigated contacts between lysosomes (green) and ER (red) by live-cell microscopy (Fig. S4, F and G), which were altered by mutant Drp1(K38A) but not by TBC1D15(D397A) or Fis1(LA) (Fig. S4, H–J). Thus, ER contacts with mitochondria and lysosomes were not ubiquitously disrupted by TBC1D15, Fis1, and Drp1 mutants, suggesting that the misregulation of mitochondrial and lysosomal dynamics observed is not solely dependent on defects in ER contact sites.

Moreover, we investigated whether the machinery regulating Drp1 GTP hydrolysis on the mitochondria was required for the regulation of mitochondrial and lysosomal tethering. As we previously showed that loss of Fis1 disrupted mitochondria–lysosome contacts (Wong et al., 2018), we examined whether it might also misregulate inter-lysosomal contacts. Indeed, loss of Fis1 led to increased and prolonged inter-lysosomal tethering (WT vs. Fis1^−/−^; Fig. S2, K and L). In addition, we found that loss of Mid51 also disrupted mitochondria–lysosome contact dynamics (WT vs. Mid51^−/−^; Fig. S2, M and N), as well as inter-lysosomal tethering (Fig. S2, O and P). Moreover, loss of either Drp1 or Mff also misregulated both mitochondria–lysosome contact dynamics (WT vs. Drp1^−/−^ vs. Mff^−/−^; Fig. S2, Q and R) and inter-lysosomal dynamics (Fig. S2, S and T), further supporting a role for properly regulated Drp1 GTP hydrolysis in modulating mitochondrial and lysosomal tethering dynamics.

### Distinct mutations in Mid51 differentially modulate Fis1 oligomerization

**Figure S5.**
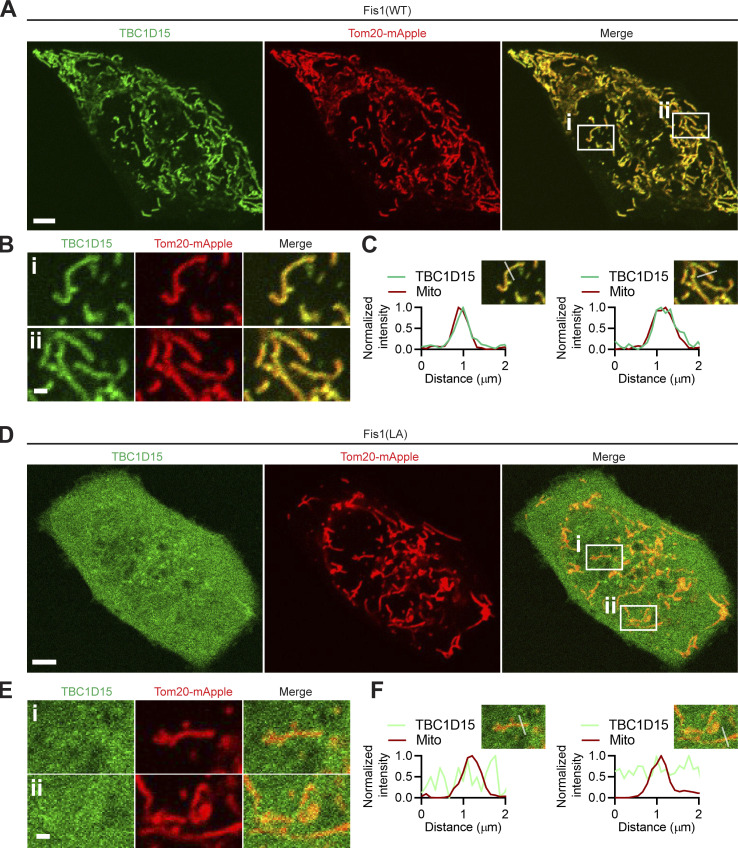
**Fis1 oligomerization regulates TBC1D15 recruitment to mitochondria. (A–C)** Confocal microscopy representative images of TBC1D15 recruitment to mitochondria in HeLa cells expressing Fis1(WT) (TBC1D15, green [YFP-TBC1D15]; mitochondria, red [Tom20-mApple]). Inset in B, corresponding linescans in C. Scale bars, 5 μm (A); 1 μm (B). **(D–F)** Confocal microscopy representative images of TBC1D15 in the cytosol in HeLa cells expressing Fis1(LA) oligomerization mutant (TBC1D15, green [YFP-TBC1D15]; mitochondria, red [Tom20-mApple]). Inset in E, corresponding linescans in F. Scale bars, 5 μm (D); 1 μm (E).

To further elucidate whether Mid51 might modulate lysosomal tethering dynamics through its regulation of Fis1 oligomerization in a Mid51/Fis1 complex, we examined whether specific mutations in Mid51 might alter Fis1 oligomerization and lysosomal tethering. We first confirmed that WT Fis1 efficiently recruited TBC1D15 to the outer mitochondrial membrane (Fig. S5, A–C), while the Fis1(LA) mutant, which cannot oligomerize (Fig. 4 I), was unable to recruit TBC1D15 to mitochondria (Fig. S5, D–F), consistent with previous studies (Onoue et al., 2013; Yamano et al., 2014; Wong et al., 2018). Thus, Fis1 oligomerization is important for recruiting TBC1D15 to mitochondria, to allow for TBC1D15 GAP activity to drive Rab7 GTP hydrolysis (Zhang et al., 2005; Peralta et al., 2010) at mitochondria–lysosome membrane contact sites (Wong et al., 2018; Kim et al., 2021).

Interestingly, mutations in distinct regions of Mid51 that mediate either its oligomerization or its binding to Drp1 were recently associated with different human diseases. The oligomerization domain mutant (Loson et al., 2014) Mid51(R169W) was recently identified as a potential candidate genetic variant for Parkinson's disease (Lubbe et al., 2020 *Preprint*), while the Drp1-binding mutant (Loson et al., 2014; Richter et al., 2014; Ma et al., 2019) Mid51(Y240N) was recently linked to dominant optic atrophy (Charif et al., 2021)*.* However, whether and how these different roles of Mid51 differentially affect Fis1 oligomerization and lysosomal network dynamics in disease has never been studied.

**Figure 7.**
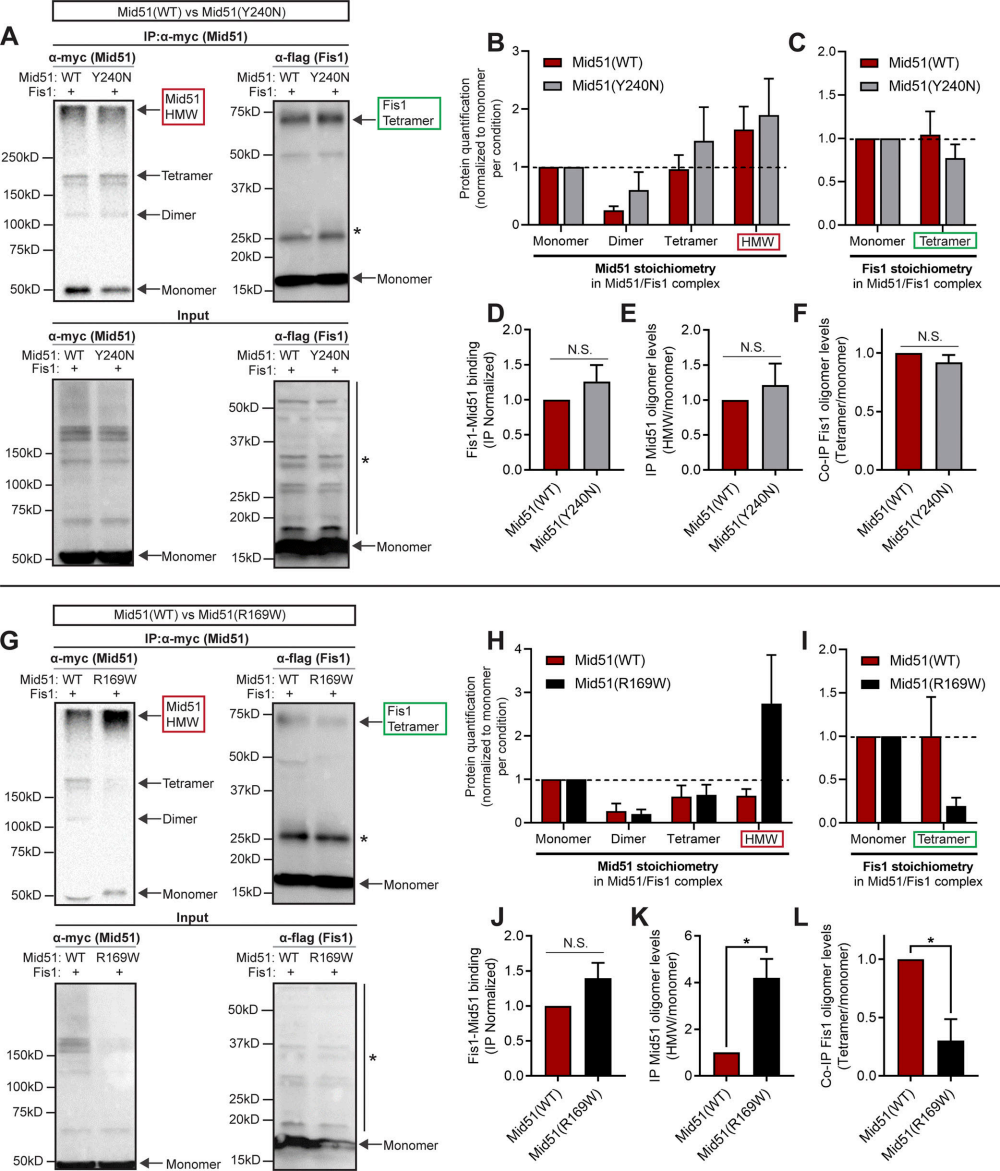
**Distinct Mid51 mutants differentially modulate Fis1 oligomerization. (A)** IP of myc-tagged Mid51(WT) or Drp1-binding domain mutant Mid51(Y240N) and co-IP of Flag-tagged Fis1, with corresponding input. *, Nonspecific bands. **(B and C)** Protein quantification and stoichiometry of the immunoprecipitated mitochondrial Mid51/Fis1 complex with Mid51(WT) or Mid51(Y240N) revealing Mid51 species (monomer, dimer, tetramer, and HMW; B) and Fis1 species (monomer, tetramer; C), normalized to monomer levels per condition, quantified from IP immunoblot (*n* = 3 independent experiments). **(D–F)** Quantification showing Drp1-binding domain mutant Mid51(Y240N) does not disrupt Fis1 oligomerization in a Mid51/Fis1 complex. Mid51(Y240N) shows normal Mid51/Fis1 binding (Fis1 IP monomer/Mid51 IP monomer ratio; D), Mid51 oligomerization (Mid51 IP [HMW/monomer ratio]; E), and Fis1 oligomerization (Fis1 IP [tetramer/monomer ratio]; F); quantified from IP immunoblot; *n* = 3 independent experiments. **(G)** IP of myc-tagged Mid51(WT) or oligomerization domain mutant Mid51(R169W) and co-IP of Flag-tagged Fis1, with corresponding input. *, Nonspecific bands. **(H and I)** Protein quantification and stoichiometry of the immunoprecipitated mitochondrial Mid51/Fis1 complex with Mid51(WT) or Mid51(R169W), revealing Mid51 species (monomer, dimer, tetramer, and HMW; H) and Fis1 species (monomer, tetramer; I), normalized to monomer levels per condition, quantified from IP immunoblot (*n* = 3 independent experiments). **(J–L)** Quantification showing oligomerization domain mutant Mid51(R169W) disrupts Fis1 oligomerization in a Mid51/Fis1 complex. Mid51(R169W) shows normal Mid51/Fis1 binding (Fis1 IP monomer/Mid51 IP monomer ratio; J), increased Mid51 oligomerization (Mid51 IP [HMW/monomer ratio]; K), and decreased Fis1 oligomerization (Fis1 IP [tetramer/monomer ratio]; L); quantified from IP immunoblot; *n* = 3 independent experiments. Mean ± SEM; unpaired two-tailed *t* test (D–F and J–L); N.S., not significant (D–F and J); *, P = 0.017 (K); *, P = 0.021 (L). Source data are available for this figure: SourceData F7.

We first examined the effect of these two distinct disease-associated Mid51 mutations on the Mid51/Fis1 oligomerization complex. The Drp1-binding mutant Mid51(Y240N) linked to dominant optic atrophy was still able to interact with WT Fis1 in a Mid51(Y240N)/Fis1 complex (Fig. 7 A). Protein quantification of the Mid51(Y240N)/Fis1 complex revealed Mid51(Y240N) present as monomer, dimer, tetramer, and HMW species (Mid51 IP; Fig. 7 B) and Fis1 as monomer and tetramer species (Fis1 co-IP; Fig. 7 C), consistent with what we observed for WT Mid51. Fis1 was still able to coimmunoprecipitate with Mid51(Y240N) at levels similar to those with WT Mid51 (Fig. 7 D), and Mid51(Y240N) showed normal Mid51 oligomerization (Charif et al., 2021) compared with WT Mid51 (Mid51 IP [HMW/monomer]; Fig. 7 E). Moreover, Mid51(Y240N) did not disrupt Fis1 oligomerization compared with WT Mid51 (Fis1 co-IP [tetramer/monomer]; Fig. 7 F), suggesting that the coupled Mid51/Fis1 oligomeric complex is not misregulated by the Drp1-binding mutant Mid51(Y240N) (Model—Mid51 mutants; Fig. S8 C, left).

We next examined whether the oligomerization domain mutant Mid51(R169W) potentially linked to Parkinson’s disease was able to disrupt Fis1 oligomerization. Mid51(R169W) was still able to interact with WT Fis1 in a Mid51(R169W)/Fis1 complex (Fig. 7, G–I), and Fis1 was still able to coimmunoprecipitate with Mid51(R169W) at levels similar to those with WT Mid51 (Fig. 7 J). However, Mid51(R169W) exhibited strikingly elevated Mid51 oligomers (Lubbe et al., 2020 *Preprint*) compared with WT Mid51 (Mid51 IP [HMW/monomer]; *, P < 0.05; Fig. 7 K). Moreover, this resulted in significantly decreased Fis1 oligomerization (Fis1 IP [tetramer/monomer]; *, P < 0.05; Fig. 7 L), demonstrating that Mid51 oligomerization regulates Fis1 oligomerization. Together, these findings suggest that WT Mid51 and Fis1 normally undergo coupled oligomerization together in a mitochondrial complex. However, if the oligomerization Mid51(R169W) mutant is already excessively oligomerized, Fis1 oligomerization cannot be coupled to the already oligomerized Mid51(R169W), leading to decreased Fis1 oligomers (Model—Mid51 mutants; Fig. S8 C, right). Thus, Mid51 and Fis1 oligomerization are dependent on each other in a tightly coupled Mid51/Fis1 complex on the mitochondria, which is selectively disrupted by the oligomerization domain mutant Mid51(R169W). In contrast, this coupled oligomerization occurs independently of Mid51 recruitment of Drp1 and is thus not affected by the Drp1-binding mutant Mid51(Y240N).

### Distinct Mid51 mutants differentially regulate lysosomal untethering and trafficking dynamics

**Figure 8.**
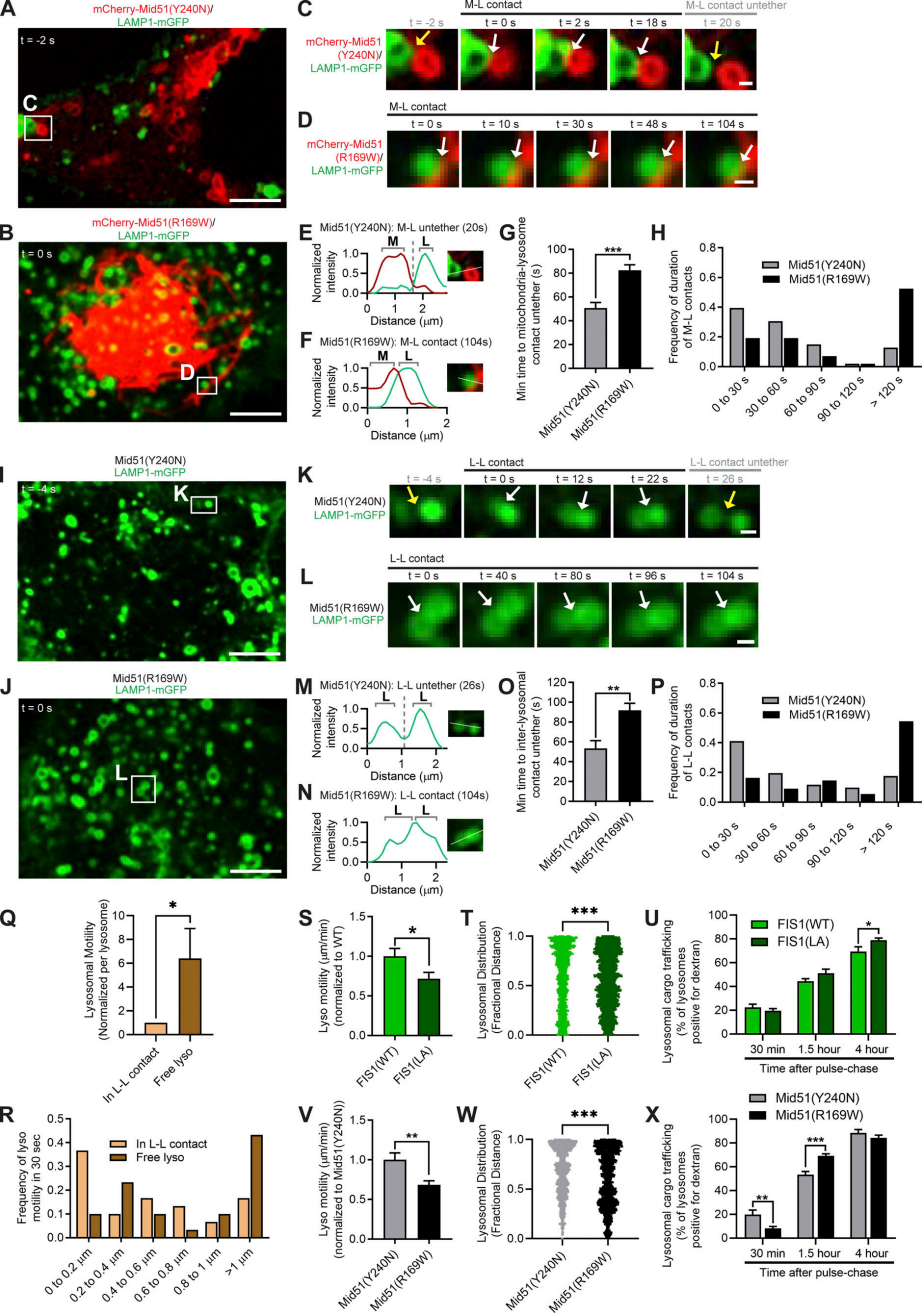
**Distinct Mid51 mutants differentially regulate lysosomal tethering and network dynamics. (A–F)** Confocal time-lapse microscopy showing prolonged mitochondria-lysosomal (M-L) tether formation (white arrows; insets in C and D) with oligomerization domain mutant Mid51(R169W) in live HeLa cells compared with Drp1-binding domain mutant Mid51(Y240N) (mitochondria mCherry-Mid51(Y240N) or mCherry-Mid51(R169W), lysosome LAMP1-mGFP). Corresponding linescans show M-L untethering for Mid51(Y240N) (E) vs. prolonged tethering for Mid51(R169W) (F). Scale bars, 5 μm (A and B); 0.5 μm (C and D). Video 9 corresponds to D. **(G and H)** Quantification and histogram of prolonged mitochondria–lysosome (M-L) tethering duration for Mid51(R169W); *n* = 101 events from 18 cells (Mid51(Y240N)); *n* = 99 events from 15 cells (Mid51(R169W)). **(I–N)** Confocal time-lapse microscopy showing prolonged inter-lysosomal (L-L) tether formation (white arrows; insets in K and L) with oligomerization domain mutant Mid51(R169W) in live HeLa cells compared with dominant optic atrophy mutant Mid51(Y240N) (lysosome LAMP1-mGFP). Corresponding linescans show M-L untethering for Mid51(Y240N) (M) vs. prolonged tethering for Mid51(R169W) (N). Scale bars, 5 μm (I and J); 0.5 μm (K and L). Video 10 corresponds to L. **(O and P)** Quantification and histogram of prolonged L-L tethering duration for Mid51(R169W); *n* = 51 events from 19 cells (Mid51(Y240N)); *n* = 55 events from 13 cells (Mid51(R169W)). **(Q and R)** Quantification and corresponding histogram showing the motility of individual lysosomes is increased after an untethering event (Free lyso) compared with while in an inter-lysosomal tether (In L-L contact; *n* = 18 events from 7 cells). **(S–U)** Quantification showing Fis1 (LA) oligomerization mutant disrupts lysosomal motility (S), lysosomal distribution (T), and lysosomal cargo trafficking dynamics (U). S, *n* = 82 events from 16 cells (Fis1(WT)); *n* = 79 events from 17 cells (Fis1(LA)); T, *n* = 2,994 lysosomes from 20 cells (Fis1(WT)); *n* = 4,141 lysosomes from 20 cells (Fis1(LA)); U, *n* = 20 cells (30 min), 20 cells (1.5 h), 19 cells (4 h; Fis1(WT)); *n* = 18 cells (30 min), 18 cells (1.5 h), 18 cells (4 h; Fis1(LA)). **(V–X)** Quantification showing Mid51(R169W) oligomerization mutant disrupts lysosomal motility (V), lysosomal distribution (W), and lysosomal cargo trafficking dynamics (X). V, *n* = 79 events from 17 cells (Mid51(Y240N); *n* = 74 events from 16 cells (Mid51(R169W)); W, *n* = 1,846 lysosomes from 15 cells (Mid51(Y240N)); *n* = 2,191 lysosomes from 15 cells (Mid51(R169W)); X, *n* = 12 cells (30 min), 15 cells (1.5 h), 13 cells (4 h; Mid51(Y240N)); *n* = 25 cells (30 min), 25 cells (1.5 h), 21 cells (4 h; Mid51(R169W)). Mean ± SEM; unpaired two-tailed *t* test (G, O, and Q–X); ***, P < 0.001 (G,T,W, and X); **, P = 0.002 (O); *, P = 0.0383 (Q); *, P = 0.0281 (S); *, P = 0.0396 (U); **, P = 0.0027 (V); **, P = 0.0021 (X).

**Figure S6.**
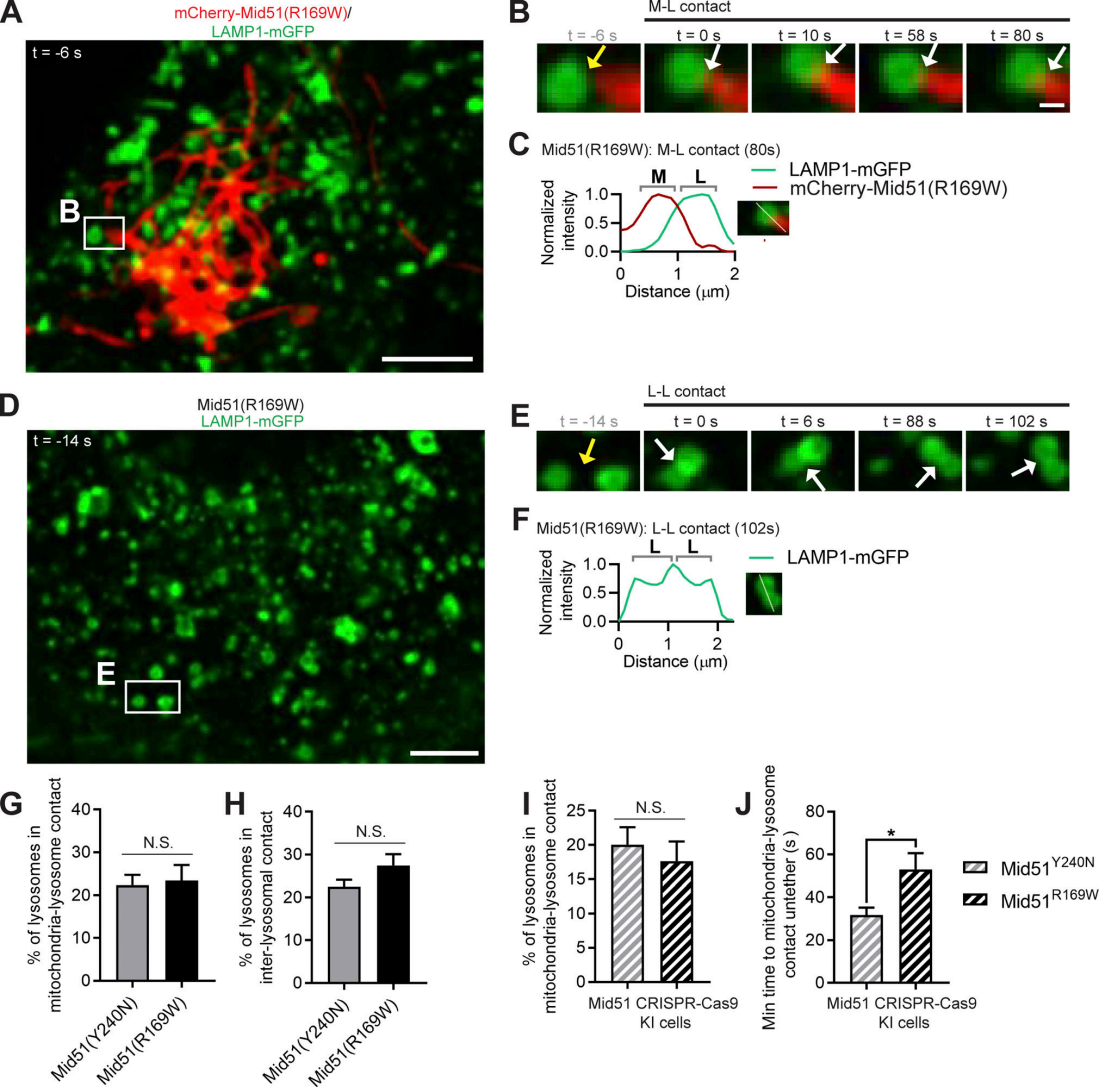
**Mid51 oligomerization domain mutant preferentially disrupts mitochondrial and lysosomal untethering dynamics. (A–C)** Confocal time-lapse microscopy of mitochondria–lysosome (M-L) tethering (white arrows; inset in B) with corresponding linescan (C) showing prolonged M-L tethering duration by oligomerization domain mutant Mid51(R169W) in live HeLa cells (mitochondria mCherry-Mid51(R169W), lysosome LAMP1-mGFP). Scale bars, 5 μm (A); 0.5 μm (B). **(D–F)** Confocal time-lapse microscopy of inter-lysosomal (L-L) tethering (white arrows; inset in E) with corresponding linescan (F) showing prolonged L-L tethering duration by Mid51(R169W) in live HeLa cells (lysosome LAMP1-mGFP). Scale bars, 5 μm (D); 0.5 μm (E). **(G and H)** Quantification of percentage of lysosomes in mitochondria–lysosome (M-L) tethers (G) and L-L tethers (H) in Mid51(Y240N) and Mid51(R169W) conditions in live HeLa cells; *n* = 19 cells (Mid51(Y240N)); *n* = 15 cells (Mid51(R169W)). **(I and J)** Quantification in CRISPR-Cas9 genetically edited HCT116 mutant Mid51 cells of percentage of lysosomes in mitochondria–lysosome (M-L) tethers; I, *n* = 21 cells (Mid51(Y240N)); *n* = 18 cells (Mid51(R169W)); prolonged mitochondria–lysosome (M-L) tether duration by Mid51(R169W)); J; *n* = 38 events from 16 cells (Mid51(Y240N)); *n* = 36 events from 17 cells (Mid51(R169W)). Mean ± SEM; unpaired two-tailed *t* test (G–J); N.S., not significant (G–I); *, P = 0.019 (J).

Finally, we investigated whether these distinct disease-associated Mid51 mutations in its oligomerization domain versus its Drp1-binding domain might differentially modulate lysosomal network dynamics. In particular, we hypothesized that the oligomerization domain mutant Mid51(R169W), which decreased Fis1 oligomerization, would preferentially result in impaired lysosomal untethering events. Indeed, mitochondria–lysosome tethers were significantly prolonged over time by oligomerization domain mutant Mid51(R169W) (Fig. 8, A–F; Fig. S6, A–C; and Video 9; white arrows in Fig. 8 D) compared with Drp1-binding mutant Mid51(Y240N) (yellow arrow; untethering in Fig. 8 C) and resulted in increased mitochondria–lysosome tethering duration (*n* > 98 events per condition; ***, P < 0.001; Fig. 8, G and H), but did not disrupt the initial formation of mitochondria–lysosome tethers (Fig. S6 G). Importantly, inter-lysosomal tethers were also prolonged over time by Mid51(R169W) (Fig. 8, I–N; Fig. S6, D–F; and Video 10; white arrows in Fig. 8 L) compared with Mid51(Y240N) (yellow arrows; untethering in Fig. 8 K). Further quantitative analysis revealed that Mid51(R169W) significantly increased inter-lysosomal tethering duration compared with Mid51(Y240N) (*n* > 50 events per condition; **, P < 0.01; Fig. 8, O and P) but did not disrupt the initial formation of tethers (Fig. S6 H). Moreover, CRISPR-Cas9 genetically edited knock-in expression of endogenous Mid51 mutations further confirmed that Mid51(R169W) preferentially disrupted mitochondrial and lysosomal tethering duration compared with Mid51(Y240N; Fig. S6, I and J). Thus, the oligomerization domain mutant Mid51(R169W) potentially linked to Parkinson’s disease, but not the Drp1-binding domain mutant Mid51(Y240N) linked to dominant optic atrophy, preferentially disrupts mitochondria–lysosome and inter-lysosomal untethering events.

**Video 9.** **Live-cell microscopy of oligomerization mutant Mid51(R169W) prolonging mitochondria–lysosome tethering.** Confocal time-lapse microscopy of prolonged mitochondria–lysosome tethering in oligomerization mutant Mid51(R169W) condition in a live HeLa cell expressing Lamp1-mGFP (lysosome; green) and mCherry-Mid51(R169W) (mitochondria; red). Video was acquired at 1 frame/2 s for 104 s and played back at 12 frames/s (24× speed). Video corresponds to Fig. 8 D. Scale bar, 0.5 µm.

**Video 10.** **Live-cell microscopy of oligomerization mutant Mid51(R169W) prolonging inter-lysosomal tethering.** Confocal time-lapse microscopy of prolonged inter-lysosomal tethering in oligomerization mutant Mid51(R169W) condition (mCherry-Mid51(R169W)) in a live HeLa cell expressing Lamp1-mGFP (lysosome; green). Video was acquired at 1 frame/2 s for 104 s and played back at 12 frames/s (24× speed). Video corresponds to Fig. 8 L. Scale bar, 0.5 µm.

**Figure S7.**
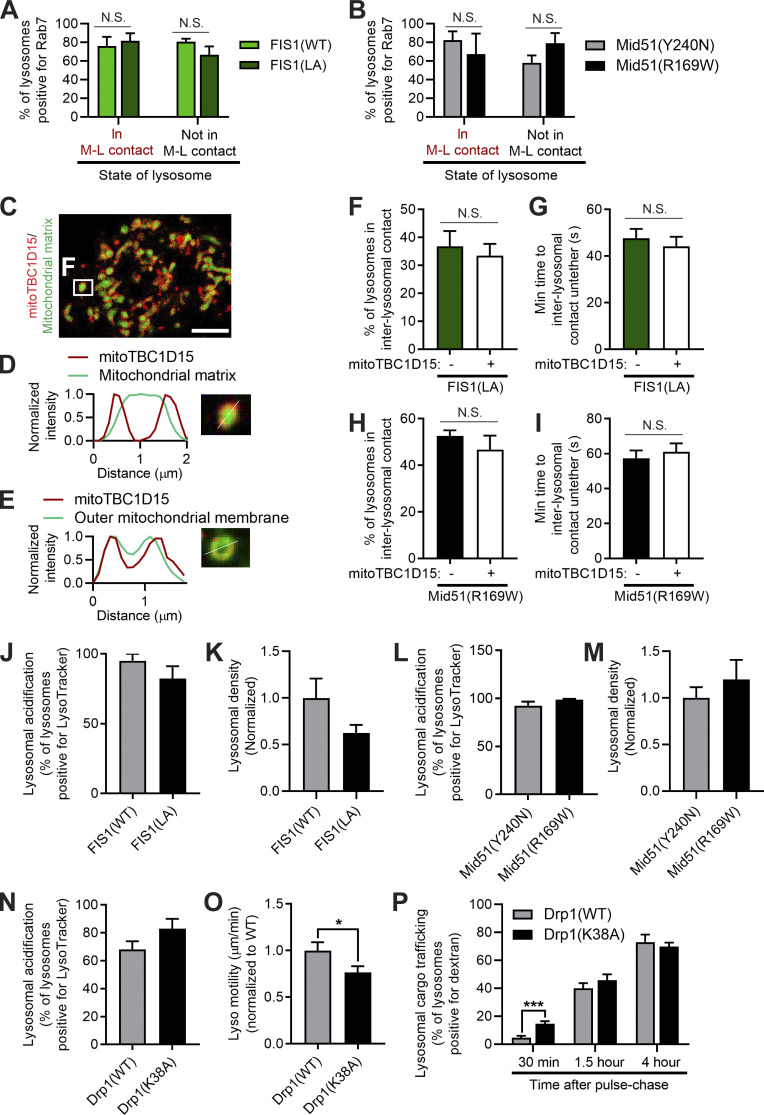
**Rab7 and TBC1D15 localization and regulation of lysosomal networks by Fis1 and Mid51. (A)** Quantification of lysosomes in mitochondria–lysosome (M-L) tether (left) or not in M-L tether (right) for the percentage of lysosomes positive for Rab7 in Fis1(WT) and Fis1(LA) conditions in live HeLa cells; *n* = 181 total lysosomes from 19 cells (Fis1(WT)); *n* = 170 total lysosomes from 13 cells (Fis1(LA)). **(B)** Quantification of lysosomes in mitochondria–lysosome (M-L) tether (left) or not in M-L tether (right) for the percentage of lysosomes positive for Rab7 in Mid51(Y240N) and Mid51(R169W) conditions in live CRISPR-Cas9 genetically edited HCT116 mutant Mid51 cells; *n* = 55 total lysosomes from 10 cells Mid51(Y240N)); *n* = 71 total lysosomes from 13 cells (Mid51(R169W)). **(C–E)** Localization of mitochondrial-targeted TBC1D15 in live HeLa cells (mCherry-tagged mitoTBC1D15 artificially targeted to the outer mitochondrial membrane via the TOM20 transmembrane domain) showing localization around the mitochondrial matrix (mEmerald-mito) with corresponding linescan (D), and linescan showing colocalization with the outer mitochondrial membrane (mEmerald-TOM20; E). **(F and G)** Quantification of percentage of lysosomes in inter-lysosomal (L-L) tether (F) and L-L tether duration (G) in Fis1(LA) live HeLa cells with or without mitoTBC1D15 (*n* = 75 events from 15 cells [− mitoTBC1D15]; *n* = 75 events from 15 cells [+ mitoTBC1D15]). **(H and I)** Quantification of percentage of lysosomes in L-L tether (H) and L-L tether duration (I) in live CRISPR-Cas9 genetically edited HCT116 mutant Mid51(R169W) cells with or without mitoTBC1D15 (*n* = 75 events from 15 cells [− mitoTBC1D15]; *n* = 75 events from 15 cells [+ mitoTBC1D15]). **(J and K)** Fis1(LA) oligomerization mutant does not regulate lysosomal acidification (percentage of lysosomes positive for LysoTracker; J) or lysosomal density (normalized to Fis1(WT); K). J, *n* = 20 cells (Fis1(WT)), *n* = 18 cells (Fis1(LA)); K, *n* = 15 cells (Fis1(WT)), *n* = 15 cells (Fis1(LA)). **(L and M)** Mid51(R169W) oligomerization mutant does not regulate lysosomal acidification (percentage of lysosomes positive for LysoTracker; L) or lysosomal density (normalized to Mid51(Y240N); M). L, *n* = 21 cells (Mid51(Y240N)); *n* = 25 cells (Mid51(R169W)); M, *n* = 15 cells (Mid51(Y240N)), *n* = 15 cells (Mid51(R169W)). **(N–P)** Quantification showing Drp1(K38A) mutant does not regulate lysosomal acidification (N) but disrupts lysosomal motility (O) and lysosomal cargo trafficking dynamics (P). N, *n* = 26 cells (Drp1(WT)); *n* = 19 cells (Drp1(K38A)); O, *n* = 73 events from 16 cells (Drp1(WT)); *n* = 74 events from 16 cells (Drp1(K38A)); P, *n* = 21 cells (30 min), 17 cells (1.5 h), 15 cells (4 h; Drp1(WT)); *n* = 17 cells (30 min), 23 cells (1.5 h), 16 cells (4 h; Drp1(K38A)). Mean ± SEM; unpaired two-tailed *t* test (A, B, and F–P); N.S., not significant (A, B, and F–N); *, P = 0.037 (O); ***, P < 0.001 (P).

Of note, the recruitment of Rab7 to lysosomes was not affected by Fis1 oligomerization mutant (Fis1(LA)) and did not depend on whether lysosomes were tethered to mitochondria (Fig. S7 A; left: in M-L contact; right: not in M-L contact). Similarly, Mid51(R169W) also did not disrupt Rab7 recruitment to lysosomes and was independent of whether lysosomes were tethered to mitochondria (Fig. S7 B; left: in M-L contact; right: not in M-L contact). In addition, regulation of lysosomal dynamics was preferentially dependent on Fis1 binding to TBC1D15, as artificial mitochondrial targeting of TBC1D15 (mitoTBC1D15) to the outer mitochondrial membrane (Fig. S7, C–E) was not sufficient to rescue inter-lysosomal tethering dynamics in either Fis1 oligomerization mutant conditions (Fig. S7, F and G) or oligomerization domain Mid51(R169W) mutant conditions (Fig. S7, H and I).

Finally, we investigated how prolonged inter-lysosomal tethering might alter lysosomal physiology. We first examined whether prolonged lysosomal tethering might disrupt the dynamics of individual lysosomes. Indeed, when we compared the motility of individual lysosomes in inter-lysosomal tethers (in L-L contact) with their motility after an untethering event (free lysosome), we found that lysosomal motility was significantly increased after untethering (Fig. 8, Q and R). Thus, we examined whether misregulation of lysosomal tethering by the Mid51/Fis1 oligomerization complex might have downstream consequences on the lysosomal network. Indeed, we found that the Fis1(LA) oligomerization mutant, which resulted in prolonged lysosomal tethering (Fig. 3 W), led to a significant decrease in lysosomal motility (*, P < 0.05; Fig. 8 S), as lysosomes could not efficiently untether from one another. Importantly, this resulted in lysosomes that were abnormally distributed and clustered closer to the cell center (***, P < 0.001; Fig. 8 T), and also accelerated cargo trafficking in live pulse-chase studies (Fig. 8 U), which was not due to defects in lysosomal acidification or changes in overall lysosomal density (Fig. S7, J and K). Thus, inhibition of Fis1 oligomerization directly prolongs lysosomal tethering, which has important consequences for lysosomal network dynamics.

We further examined whether the oligomerization mutant Mid51(R169W), which also led to defective Fis1 oligomerization (Fig. 7 L) and caused prolonged lysosomal tethering (Fig. 8 O), might have similar effects on disrupting lysosomal physiology. Indeed, oligomerization mutant Mid51(R169W) also led to a significant decrease in lysosomal motility (**, P < 0.01; Fig. 8 V), as lysosomes could not efficiently untether from one another, compared with the Drp1-binding mutant Mid51(Y240N), which did not disrupt Fis1 oligomerization (Fig. 7 F). Importantly, this also resulted in lysosomes that were abnormally distributed and clustered closer to the cell center (***, P < 0.001; Fig. 8 W), as well as accelerated cargo trafficking, which was not due to defects in lysosomal acidification or overall density (Fig. S7, L and M), consistent with what we observed for the Fis1(LA) oligomerization mutant. Inhibition of Drp1 GTP hydrolysis (Drp1(K38A)) also similarly led to decreased lysosomal motility and disrupted cargo trafficking dynamics (*, P < 0.05; Fig. S7, N–P). Thus, misregulation of lysosomal untethering by altering Fis1 or Mid51 oligomerization leads to defective lysosomal motility, resulting in altered lysosomal distribution and misregulation of cargo trafficking for the lysosomal network.

**Figure S8.**
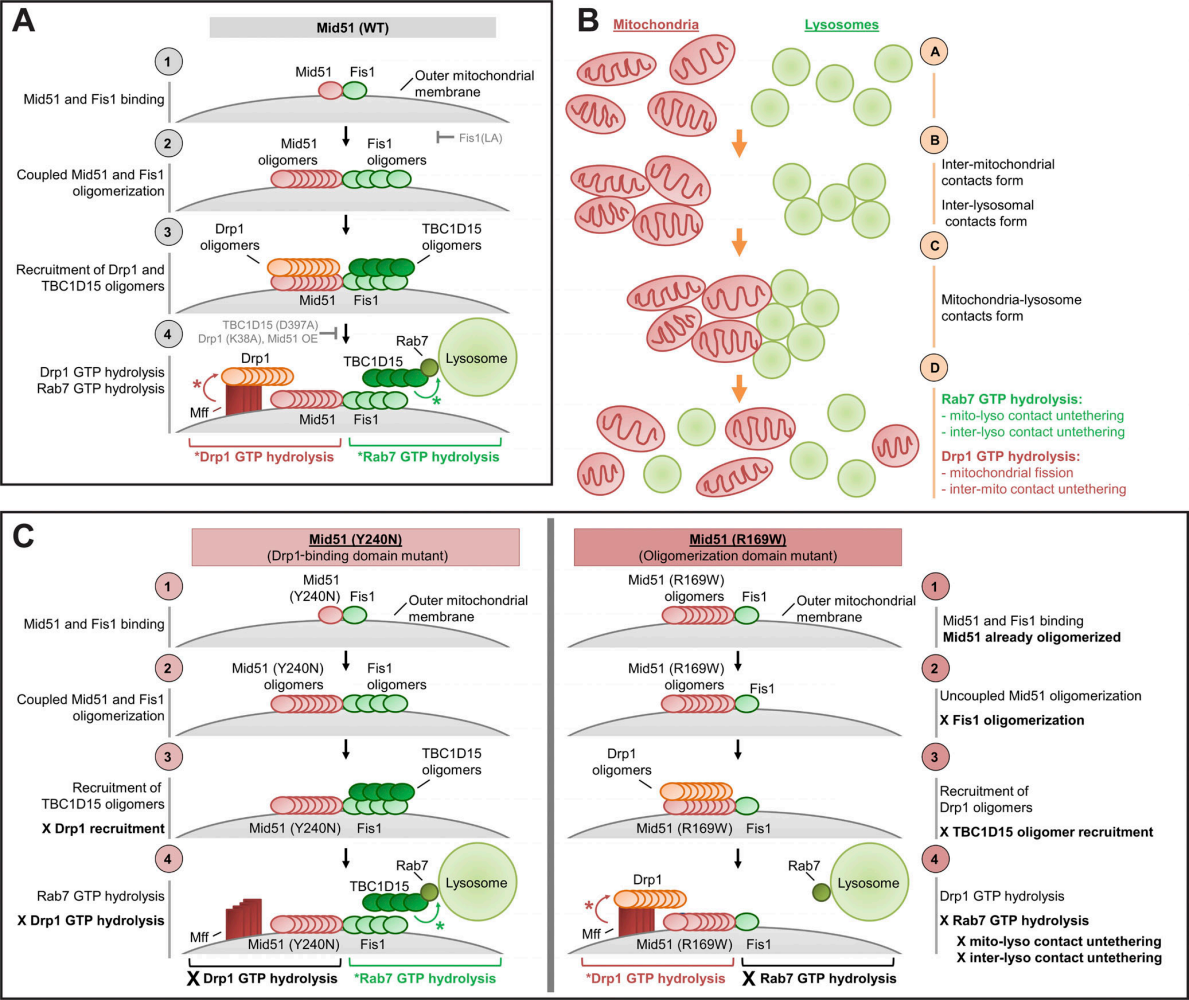
**Model of mitochondrial and lysosomal network regulation via coupled Mid51/Fis1 oligomerization complex. (A)** Model—Normal: (1 and 2) Mid51 and Fis1 undergo coupled oligomerization in a Mid51/Fis1 complex on the outer mitochondrial membrane; (3 and 4) Mid51 oligomers promote Drp1 oligomerization and subsequent Drp1 GTP hydrolysis via Mff (red arrow), while Fis1 oligomers promote TBC1D15 (Rab7-GAP) recruitment to mitochondria to drive Rab7 GTP hydrolysis at mitochondria–lysosome tethers (green arrow). Inhibiting either Mid51/Fis1 oligomerization (Fis1(LA)), Rab7 GTP hydrolysis (TBC1D15(D397A)), or Drp1 GTP hydrolysis (Drp1(K38A); Mid51) disrupts this pathway (grey arrows). See Discussion for details. **(B)** Rab7 GTP hydrolysis promotes mitochondria–lysosome and inter-lysosomal contact untethering, while Drp1 GTP hydrolysis promotes mitochondrial fission and inter-mitochondrial untethering, resulting in untethering events which help redistribute both mitochondrial and lysosomal networks. **(C)** Model—Mid51 mutants); left: Mid51(Y240N) Drp1-binding domain mutant which is linked to dominant optic atrophy disrupts Drp1 recruitment but not Mid51 oligomerization, leading to the selective inhibition of Drp1 GTP hydrolysis; right: Mid51(R169W) oligomerization domain mutant which is potentially linked to Parkinson’s disease disrupts Mid51 oligomerization but not Drp1 recruitment, leading to uncoupled and defective Fis1 oligomerization and the selective inhibition of Rab7 GTP hydrolysis, resulting in the misregulation of lysosomal tethering dynamics.

Altogether, our study identifies a novel mitochondrial Fis1/Mid51 oligomerization complex that controls lysosomal untethering. Distinct disease-associated Mid51 mutations in either its oligomerization domain or Drp1-binding domain differentially target Mid51/Fis1 oligomerization, which regulates Rab7-GTP hydrolysis-dependent lysosomal untethering events to modulate the overall lysosomal network (Model—Mid51 mutants; Fig. S8 C).

## Discussion

Elucidating the specific machinery involved in reorganizing lysosomal networks in living cells is key for advancing our understanding of cell biology. While GTP-bound Rab7 was previously known to promote late endosomal or lysosomal tethering before fusion events (Langemeyer et al., 2018), we now demonstrate that mitochondria can further modulate lysosomal networks by driving lysosomal untethering events via Rab7 GTP hydrolysis, mediated by Fis1 oligomerization and recruitment of TBC1D15 (Rab7-GAP) to mitochondria.

Rab7 GTP hydrolysis regulates lysosomal dynamics (Langemeyer et al., 2018), and Drp1 GTP hydrolysis regulates mitochondrial fission (Smirnova et al., 2001), but whether these two key GTPase pathways mechanistically converge was previously unknown. Our findings highlight a potential model for the convergence of these two pathways via a Mid51/Fis1 oligomeric complex on the mitochondria, which couples the machinery required for downstream Drp1 and Rab7 GTP hydrolysis steps (Model; Fig. S8 A): (1 and 2), Mid51 and Fis1 interact on the outer mitochondrial membrane and promote each other’s oligomerization; (3), Mid51 oligomers subsequently recruit and mediate Drp1 oligomerization on mitochondria (Koirala et al., 2013; Loson et al., 2014; Kalia et al., 2018), while Fis1 oligomers recruit TBC1D15 to mitochondria (Jofuku et al., 2005; Onoue et al., 2013), (4), TBC1D15 on mitochondria drives Rab7 GTP hydrolysis on lysosomes (green arrow, right; Zhang et al., 2005; Peralta et al., 2010; Wong et al., 2018), while Mff binds oligomeric Drp1 to drive Drp1 GTP hydrolysis on mitochondria (red arrow, left; Gandre-Babbe and van der Bliek, 2008; Otera et al., 2010; Koirala et al., 2013; Clinton et al., 2016; Macdonald et al., 2016; Osellame et al., 2016; Zhang et al., 2016; Yu et al., 2017). Importantly, Rab7 GTP hydrolysis mediates both mitochondria–lysosome (Wong et al., 2018, 2019a; Cisneros et al., 2022) and inter-lysosomal untethering events, which we found were tightly temporally linked, while Drp1 GTP hydrolysis promotes mitochondrial fission (Smirnova et al., 2001) and inter-mitochondrial untethering events (Wong et al., 2019b). This mechanistic coupling of both Rab7 and Drp1 GTP hydrolysis machinery may facilitate the dynamic reorganization of tethered lysosomal/mitochondrial networks over time (Fig. S8 B) and may be additionally modulated by specific Drp1 isoforms (Macdonald et al., 2016; Itoh et al., 2018).

Moreover, inhibiting either Mid51/Fis1 oligomerization or downstream Drp1/Rab7 GTP hydrolysis (gray arrows; Fig. S8 A) is sufficient to disrupt both inter-lysosomal and mitochondria–lysosome untethering events (Wong et al., 2018) regulated by Rab7 GTP hydrolysis. Notably, inhibiting these steps also disrupts mitochondrial fission (Wong et al., 2018) and inter-mitochondrial untethering events (Wong et al., 2019b), which are regulated by Drp1 GTP hydrolysis, further suggesting a functional convergence of these two pathways. While yeast yFis1 recruits Drp1 oligomers to mitochondria via cytosolic adaptors (Mozdy et al., 2000; Tieu et al., 2002; Griffin et al., 2005), the role of mammalian Fis1 in this pathway has long been elusive. Our findings demonstrate a potential role for mammalian Fis1, through its binding to Mid51 in a coupled Mid51/Fis1 oligomeric mitochondrial complex. Importantly, we show that Fis1 oligomers directly promote the oligomerization of the mitochondrial adaptor Mid51, which is important for driving Drp1 oligomerization on mitochondria required for subsequent Drp1 GTP hydrolysis (Koirala et al., 2013; Loson et al., 2014; Kalia et al., 2018).

Finally, we found that distinct disease-associated Mid51 mutations can uncouple this pathway to selectively disrupt opposite arms of the pathway and drive the pathogenesis of two distinct human diseases (Model—Mid51 mutants; Fig. S8 C). Dominant optic atrophy is an inherited neuropathy characterized by degeneration of the optic nerves (Yu-Wai-Man et al., 2014). Mutant Mid51(Y240N) was recently linked to dominant optic atrophy (Charif et al., 2021) and is located in Mid51’s Drp1-binding region, which led to defective mitochondrial fission/fusion dynamics (Charif et al., 2021) that are dependent on Drp1 GTP hydrolysis, but normal Mid51/Fis1 oligomerization and lysosomal networks. Conversely, Mid51(R169W) is a potential candidate genetic variant for Parkinson’s disease (Lubbe et al., 2020 *Preprint*), a movement disorder caused by loss of dopaminergic neurons (Poewe et al., 2017). Mid51(R169W) is located in Mid51’s oligomerization domain and selectively disrupted the coupling of Mid51/Fis1 oligomerization, which led to defective lysosomal network dynamics that are dependent on Rab7 GTP hydrolysis, but normal mitochondrial fission/fusion dynamics (Lubbe et al., 2020 *Preprint*). Thus, different mutations in a single mitochondrial protein selectively uncouple Drp1 GTP hydrolysis from Rab7 GTP hydrolysis in opposing directions, resulting in specific deficits in mitochondrial versus lysosomal dynamics.

In summary, this work provides new insights into the modulation of lysosomal dynamics by an oligomeric Mid51/Fis1 complex on the mitochondria, and further underscores the importance of this pathway in health and disease.

## Materials and methods

### Reagents

The following plasmids were obtained from Addgene: LAMP1-mGFP was a gift from Esteban Dell’Angelica (#34831; Addgene; http://n2t.net/addgene:34831; RRID:Addgene_34831; Falcon-Perez et al., 2005; University of California Los Angeles, Los Angeles, CA), mito-BFP (#49151; Addgene; http://n2t.net/addgene:49151; RRID:Addgene_49151) and mCh-Drp1 (#49152; Addgene; http://n2t.net/addgene:49152; RRID:Addgene_49152) were gifts from Gia Voeltz (Friedman et al., 2011; University of Colorado Boulder, Boulder, CO); EGFP-RAB7A WT was a gift from Qing Zhong (#28047; Addgene; http://n2t.net/addgene:28047; RRID:Addgene_28047; Sun et al., 2010; Shanghai Jiaotong University School of Medicine, Shanghai, China); mApple-TOMM20-N-10 (#54955; Addgene; http://n2t.net/addgene:54955; RRID:Addgene_54955), mEmerald-TOMM20-N-10 (#54282; Addgene; http://n2t.net/addgene:54282; RRID:Addgene_54282), mEmerald-Mito-7 (#54160; Addgene; http://n2t.net/addgene:54160; RRID:Addgene_54160), mCherry-ER-3 (plasmid # 55041; Addgene; http://n2t.net/addgene:55041; RRID:Addgene_55041), mEmerald-ER-5 (plasmid # 54083; Addgene; http://n2t.net/addgene:54083; RRID:Addgene_54083), and mTagBFP2-Lysosomes-20 (#55308; Addgene; http://n2t.net/addgene:55308; RRID:Addgene_55308; Subach et al., 2011) were gifts from Michael Davidson (Florida State University, Tallahassee, FL). N-terminal HA-TBC1D15 (WT and D397A mutant) and rat Flag-Fis1 (WT and LA mutant) were gifts from Naotada Ishihara (Jofuku et al., 2005; Onoue et al., 2013; Osaka University, Osaka, Japan); YFP-TBC1D15 was a gift from Richard Youle (Yamano et al., 2014; National Institutes of Health, Bethesda, MD); GFP-RAB7-Q67L was a gift from Aimee Edinger (Romero Rosales et al., 2009; University of California, Irvine, CA); and mCherry-Mff was a gift from Elena Kolobova (Mason et al., 2014; Vanderbilt University Medical Center, Nashville, TN). mCherry-Drp1(K38A), Mcherry-Mid51 (WT, R169W and Y240N mutants), myc-Mid51 (WT, R169W and Y240N mutants), human Flag-Fis1 (WT and LA mutant), mitochondrial targeted TBC1D15 (WT; mitoTBC1D15; N-terminally tagged with transmembrane domain of human TOM20 and mCherry), and bicistronic BFP + SKIP (WT; AAA mutant [aa 610–612: KMI changed to AAA]; ΔRUN mutant [N terminus aa 1-536 deleted]) were generated using VectorBuilder. The following antibodies were used for IP studies: mouse Myc-Tag antibody (9B11; 2276; Cell Signaling), rabbit Myc-tag (2272S; Cell Signaling), and rabbit Flag-tag (F7425; Sigma-Aldrich). The following reagents were used: LysoTracker Red DND-99 (L7528; Thermo Fisher Scientific), LysoTracker Green DND-26 (L7526; Thermo Fisher Scientific), Dextran cascade blue 10,000 MW (D1976; Thermo Fisher Scientific), and Dextran Alexa Fluor 568 (D22912; Thermo Fisher Scientific).

### Cell culture and transfections

HeLa cells (ATCC) were verified by cytochrome c oxidase subunit I and short tandem repeat testing and confirmed to be negative for Mycoplasma contamination. WT and Mid51^−/−^ HeLa cells were generated by Synthego Corp. HeLa cells were cultured in DMEM (11995-065; Gibco) supplemented with 10% (vol/vol) FBS, 100 units/ml penicillin, and 100 μg/ml streptomycin and maintained at 37°C in a 5% CO_2_ incubator. WT, Fis1^−/−^, Drp1^−/−^, and Mff^−^/^−^ HCT116 cells were gifts from Richard Youle (Yamano et al., 2014). CRISPR-Cas9 genetically edited knock-in mutations in Mid51 were generated in HCT116 cells harboring Mid51(Y240N/WT; Mid51^Y240N^) and Mid51(R169W/R169W; Mid51^R169W^) mutations (Synthego Corp). HCT116 cells were cultured in McCoy’s 5A with L-glutamine (ATCC 30-2007) supplemented with 10% (vol/vol) FBS, 100 units/ml penicillin, 100 μg/ml streptomycin, and nonessential amino acids. HCT116 Mid51 mutant cells were analyzed for mitochondria-lysosomal tether formation and duration, the role of mitochondrial-targeted TBC1D15, lysosomal acidification, and lysosomal engulfment of cargo. Cells were transfected using Lipofectamine 2000 (Invitrogen). Dextran blue and Dextran Alexa Fluor 568 were used at 0.5 mg/ml, washed three times, and chased for 30 min or 1.5 or 4 h. LysoTracker Red was used at 2 μm (37°C, 30-min incubation and washed three times), and LysoTracker Green was used at 1 μm (37°C, 30-min incubation and washed three times before imaging).

### Confocal microscopy

Images were acquired on a Nikon A1R laser scanning confocal microscope with GaAsp detectors using a Plan Apo λ 100× 1.45-NA oil-immersion objective (Nikon) in a temperature-controlled chamber (37°C) at 5% CO_2_ using NIS-Elements (Nikon) at 1 frame every 2–3 s. Confocal microscopy experiments were conducted with fluorescent plasmids listed above, HA-tagged TBC1D15, and rat Flag-Fis1 plasmids. Dual-color videos were acquired as consecutive green–red images, and tricolor videos were acquired as consecutive green–red–blue images. Images of ER with mitochondria and lysosomes in Fis1 and Drp1 conditions were imaged on a Zeiss LSM 980 confocal microscope with GaAsp detectors using an α Plan-Apochromat 100× 1.46-NA oil DIC immersion objective (Zeiss) in a temperature-controlled chamber (37°C) at 5% CO_2_ using Zen Blue (Zeiss).

### EM

For EM, cells were grown on coverslips and fixed in a mixture of 2.5% glutaraldehyde and 2% PFA in 0.1 M cacodylate buffer for 2–24 h at 4°C. After postfixation in 1% osmium tetroxide and 3% uranyl acetate, cells were dehydrated in a series of ethanol, embedded in Epon resin, and polymerized for 48 h at 60°C. Ultrathin sections were made using a UCT ultramicrotome (Leica Microsystems) and contrasted with 4% uranyl acetate and Reynolds’s lead citrate. Samples were imaged using a Tecnai Spirit G2 transmission electron microscope (FEI) operated at 80 kV. Images were captured with an Eagle 4 k HR 200-kV CCD camera.

### SIM

SIM super-resolution images were taken on a Nikon N-SIM system with a 100× 1.49-NA oil-immersion objective lens (Nikon). Images were captured using NIS-Elements (Nikon) at 1 frame every 7 s and reconstructed using slice reconstruction in NIS-Elements (Nikon). Images for live-cell imaging (live N-SIM) were taken at a single z-plane. Cells used for live-cell imaging were maintained in a temperature-controlled chamber (37°C) at 5% CO_2_ in a TokaiHit stagetop incubator.

### IP/immunoblot

To examine Mid51 and Fis1 binding, HeLa cells were transfected for 24 h with myc-tagged human Mid51 (WT, R169W, or Y240N) and Flag-tagged human Fis1 (WT or LA). Cells were lysed in EBC buffer (C14-10; Boston Bioproducts) with cOmplete Protease Inhibitor Cocktail (11873580001; Roche) and sonicated. Lysates were immunoprecipitated for myc-Mid51 using protein G–coupled Dynabeads (100003D; Invitrogen) incubated in anti-myc antibody (mouse) and washed in EBC buffer. Samples were subsequently heated for 20 min at 55°C. Equal total protein levels of immunoprecipitate were separated by SDS-PAGE and analyzed by Western blot. Specifically, samples were loaded onto a 4–20% Tris-Glycine gel (Novex), run at 135 V for 1 h and 30 min in Tris-Glycine SDS running buffer, and transferred using Trans Blot Turbo Transfer system (Bio-Rad), and SDS-resistant complexes were imaged with Bio-Rad Image Lab according to standard protocols, using anti-myc antibody (rabbit) and anti-Flag antibody (rabbit). Fig. 7 A, lane 1 (WT Mid51 and WT Fis1), corresponds to Fig. S3 A, lane 2 (WT Mid51 and WT Fis1). Protein quantification was conducted in ImageJ (National Institutes of Health [NIH]). Protein quantification of the immunoprecipitated Mid51/Fis1 complex for Fis1 species (monomer, tetramer) and Mid51 species (monomer, dimer, tetramer, and HMW) were analyzed as the immunoprecipitated levels normalized to the immunoprecipitated monomer species; for each protein per condition). Measured band intensities for coimmunoprecipitated Fis1 oligomer (IP Fis1 [tetramer/monomer ratio]), immunoprecipitated Mid51 oligomer (IP Mid51 [HMW oligomer/monomer ratio]), and Fis1 binding to Mid51 (IP Fis1 monomer/IP Mid51 monomer) were calculated. Values were further normalized to either Mid51 (WT) or Fis1 (WT) depending on the condition. To confirm Mid51 and Fis1 binding, control IP studies were conducted with WT Fis1 ± WT Mid51, or WT Mid51 ± WT Fis1. Measured band intensities for immunoprecipitated Mid51 levels (IP Mid51 monomer) and coimmunoprecipitated Fis1 levels (IP Fis1 monomer) were calculated and normalized to the condition expressing both WT Mid51 and WT Fis1.

### Image analysis

Inter-lysosomal contacts and mitochondria–lysosome contacts imaged in live cells were categorized as those that showed the two organelles in close proximity (<0.1 µm) for >10 s in time-lapse images (Belton et al., 2022; Wong et al., 2018). The percentage of lysosomes in contacts were quantified as the percentage of lysosomes that formed contacts with mitochondria or other lysosomes in a region of interest per cell. All contacts analyzed for the minimum duration of contacts were those that had already formed at the beginning of the video. The minimum duration of contact was quantified as the time before contact termination and dissociation (organelles detaching from one another) over a 120-s video. Any contacts that lasted throughout the video and that were still in contact by the end of the video were categorized as 120 s. Lysosomal fusion events were defined as two lysosomes that fused together to become a single lysosomal vesicle. The rate of lysosomal untethering vs. lysosomal fusion events was calculated from 120-s videos for the total number of events occurring in the cell in the imaged plane of view and reported as a rate (no. events/min; analyzed per cell) and the frequency of events (percentage; analyzed per experiment). The state of inter-lysosomal contacts after 30, 60, and 120 s (remain tethered or untether), and the ultimate fate of inter-lysosomal contacts within 120 s (untether or fusion), were calculated from initial contact formation.

The expected probability that mitochondria would be at the site of an inter-lysosomal contact untethering event by random chance was calculated as the density of mApple-Tom20 in the cytosol from *n* = 28 living cells, using ImageJ (NIH). The percentage of inter-lysosomal untethering events marked by a mitochondria–lysosome contact site was calculated. The time between mitochondria–lysosome contact formation/untethering and inter-lysosome contact formation/untethering were calculated for contacts that formed with the same lysosome. The fate of inter-lysosomal contacts after 10 s (remain tethered or untether) without either lysosome being in contact with mitochondria (– Mitochondria) or upon mitochondria untethering from either one of the two lysosomes (M-L contact untether) were calculated. Analysis of mitochondria or lysosomes in contact with the ER was conducted using confocal images thresholded in ImageJ and analyzed for their Pearson correlation coefficient using the EzColocalization Plugin (ImageJ; Stauffer et al., 2018). Mitochondria (mEmerald-TOM20), lysosomes (mTagBFP2-Lysosomes-20), and ER (mCherry-ER-3) were analyzed for TBC1D15 and Fis1 conditions. Mitochondria (mito-BFP), lysosomes (mTagBFP2-Lysosomes-20), and ER (mEmerald-ER-5) were analyzed for Drp1 conditions. Inter-lysosomal formation and tethering analysis from control WT HCT116 cells were used to compare against both Fis1^−/−^ conditions, as well as Drp1^−/−^ and Mff^−/−^ conditions.

Analysis of Rab7 on lysosomes was conducted by analyzing lysosomes (mTagBFP2-lyso) that were either in contact with mitochondria (mApple-TOM20) for >10 s (in M-L contact) or not in contact with mitochondria (not in M-L contact) and quantifying the percentage of lysosomes that were positive for GFP-Rab7 localization on the lysosomal membrane, calculated per experiment. The effect of artificially targeting TBC1D15 to mitochondria (+ mitoTBC1D15) was analyzed for the percentage of lysosomes that formed contacts with other lysosomes in a region of interest per cell, and for the minimum duration of contact tethering over a 120-s video. Efficient targeting of mitoTBC1D15 in the outer mitochondrial membrane was assessed by imaging and linescan analysis for its localization around the mitochondrial matrix (mEmerald-mito) and its colocalization with the outer mitochondrial membrane (mEmerald-TOM20).

Lysosomal motility was analyzed for individual lysosomes in inter-lysosomal tethers 30 s before the inter-lysosomal untethering event (in L-L contact) compared with 30 s after the untethering event (Free lyso) and normalized per lysosome. To compare between different conditions, lysosomal motility was analyzed from 5 lysosomes/cell from >15 cells per condition, for the total displacement of each lysosomes from 180-s videos, with outliers removed per condition (3 maximum and 3 minimum) and calculated per lysosome. Lysosomal distribution (fractional distance) was analyzed as previously described (Jongsma et al., 2020): Fluorescence intensities along multiple-line regions of interest (using the line profile tool in ImageJ) were normalized to the median, and background pixels were excluded from the analysis by determining the signal threshold. Distances corresponding to the remaining pixels were plotted as fractions of the distance from the nucleus (0) to the plasma membrane (1) along the same line. Lysosomal cargo trafficking dynamics were analyzed as the percentage of lysosomes (Lamp1-mGFP) positive for Dextran blue for Fis1 and Mid51 conditions and the percentage of lysosomes (mtagBFP2-lyso) positive for Dextran Alexa Fluor 568 for Drp1 conditions after 30 min and 1.5 and 4 h of dextran pulse-chase in live cells (calculated per cell). Lysosomal density was analyzed as the percentage of the cell occupied by lysosomes (Lamp1-mGFP) in a single *z*-plane and image (calculated per cell) and normalized to the corresponding WT condition. Lysosomal acidification was analyzed as the percentage of lysosomes (Lamp1-mGFP) positive for LysoTracker Red for Fis1 and Mid51 conditions and the percentage of lysosomes (mtagBFP2-lyso) positive for LysoTracker Green for Drp1 conditions, calculated per experiment.

### Statistical analysis, graphing, and figure assembly

Data were analyzed using unpaired two-tailed Student *t* test (for two datasets) or one-way ANOVA with Tukey’s post hoc test (for multiple data sets). Fisher’s exact test was used to compare the percentage of inter-lysosomal contact untethering events with mitochondria–lysosome contacts versus the percentage expected by chance. Data presented are means ± SEM (except in histograms). All statistical tests were analyzed from *n* ≥ 3 independent experiments per condition (see figure legends for detailed events and cell numbers). Data distribution was assumed to be normal but was not formally tested. Statistical analysis of SKIP on contact formation and tethering duration is shown for SKIP mutants compared with SKIP (WT). Statistics are not shown for the detailed protein quantification of coimmunoprecipitated species. Statistics and graphing were performed using Prism software (GraphPad). Videos and images were analyzed and assembled using ImageJ (NIH). All final figures were assembled in Illustrator (Adobe).

### Online supplementary material

Fig. S1 shows EM and super-resolution imaging of inter-lysosomal tethering dynamics and regulation by mitochondria. Fig. S2 shows that Rab7 GTP hydrolysis and Drp1 GTP hydrolysis machinery regulate inter-lysosomal tethers. Fig. S3 shows that Mid51 interacts with Fis1 in a Mid51/Fis1 complex and regulates mitochondrial and lysosomal tethering. Fig. S4 shows regulation of mitochondrial and lysosomal contacts with the ER. Fig. S5 shows that Fis1 oligomerization regulates recruitment of TBC1D15 to mitochondria. Fig. S6 shows that the Mid51 oligomerization domain mutant preferentially disrupts mitochondrial and lysosomal untethering dynamics. Fig. S7 shows the role of Rab7 and TBC1D15 localization and the effect on lysosomal networks by mitochondrial proteins. Fig. S8 shows a model of mitochondrial and lysosomal network regulation via coupled Mid51/Fis1 oligomerization complex. Video 1 shows super-resolution microscopy of inter-lysosomal tethering dynamics. Video 2 shows live-cell microscopy of an inter-lysosomal untethering event. Video 3 shows live-cell microscopy of a lysosomal network cluster disassembled by multiple inter-lysosomal untethering events. Video 4 shows super-resolution SIM of an inter-lysosomal untethering event marked by mitochondria. Video 5 shows live-cell microscopy of lysosomal cluster disassembling after mitochondrial tethering. Video 6 shows live-cell microscopy of prolonged mitochondria–lysosome tethering by Mid51. Video 7 shows live-cell microscopy of mitochondria–lysosome untethering event by Mff. Video 8 shows live-cell microscopy of prolonged inter-lysosomal tethering by Mid51. Video 9 shows live-cell microscopy of oligomerization mutant Mid51(R169W) prolonging mitochondria–lysosome tethering. Video 10 shows live-cell microscopy of oligomerization mutant Mid51(R169W) prolonging inter-lysosomal tethering.

## Data availability

All data that support the findings of this study are available from the authors upon reasonable request.

## Supplementary Material

SourceData F4 is the source file for Fig. 4.

SourceData F7 is the source file for Fig. 7.

SourceData FS3 is the source file for Fig. S3.
