# Supplementary figures and images for: Mid51/Fis1 mitochondrial oligomerization complex drives lysosomal untethering and network dynamics
Source: J Cell Biol. 2022 Aug 31;221(10):e202206140. doi: 10.1083/jcb.202206140 (PMC9437119; doi:10.1083/jcb.202206140)

Figure 4E

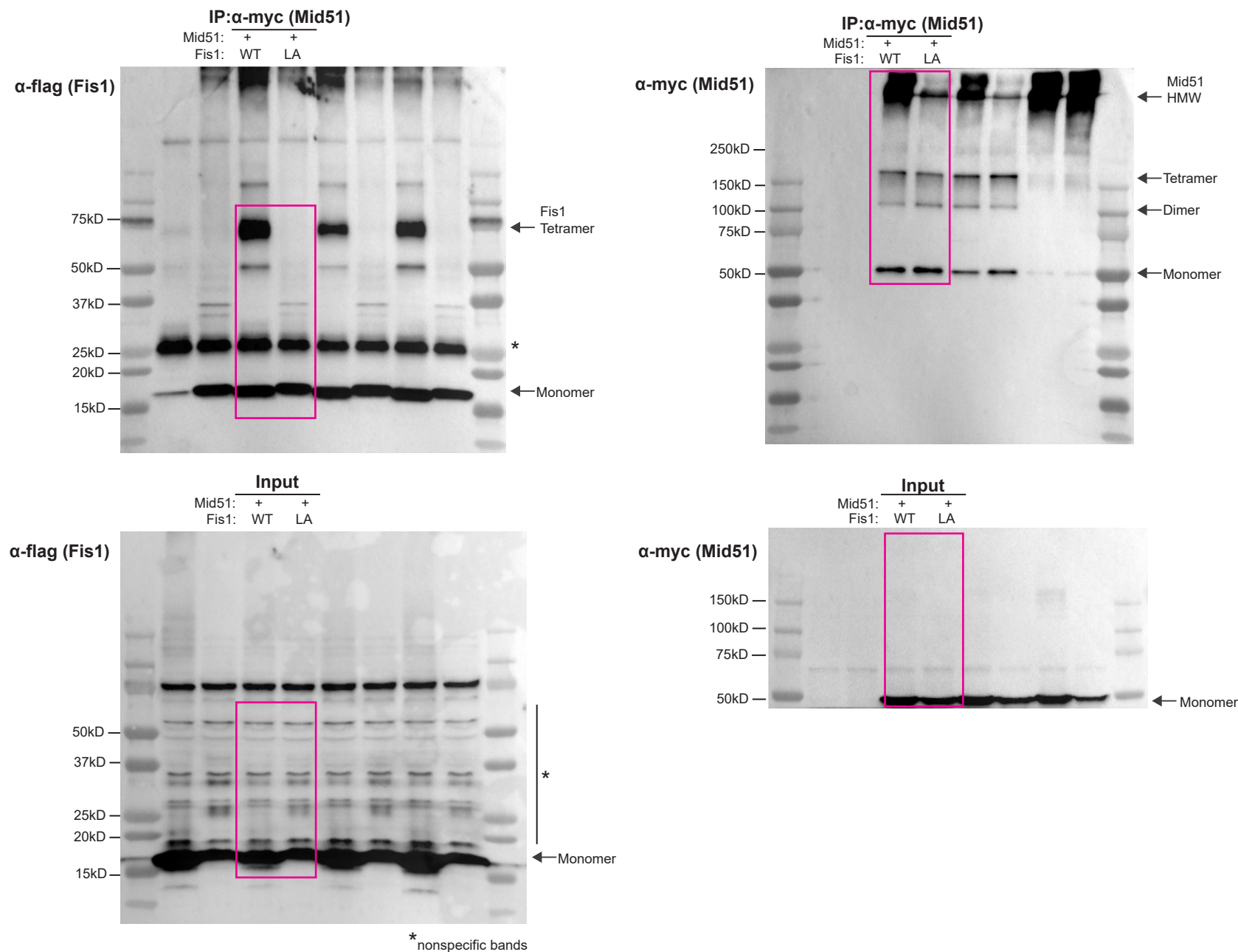

Supplement: SourceData F4 — is the source file for Fig. 4. [file JCB_202206140_SourceDataF4.pdf]

Figure 7A and 7G

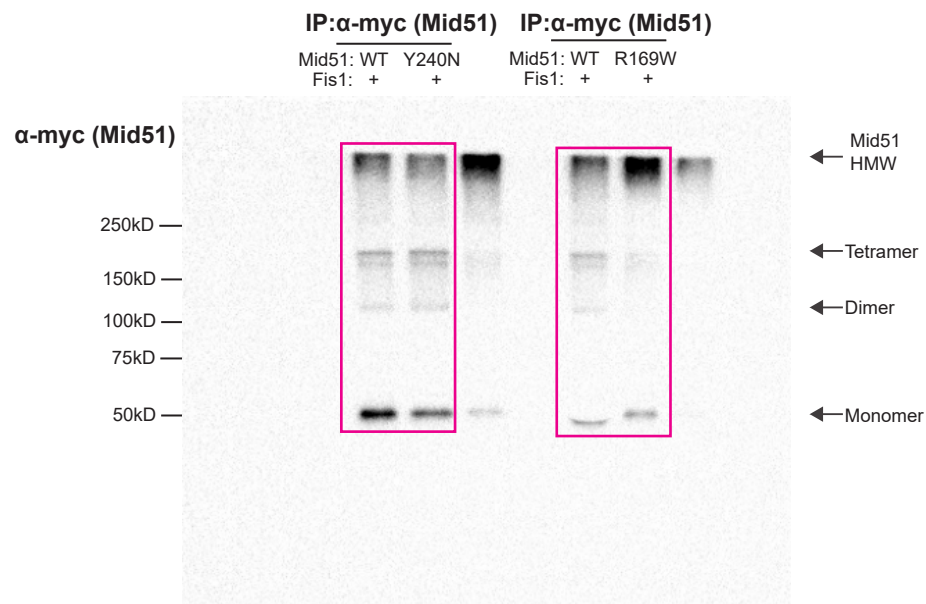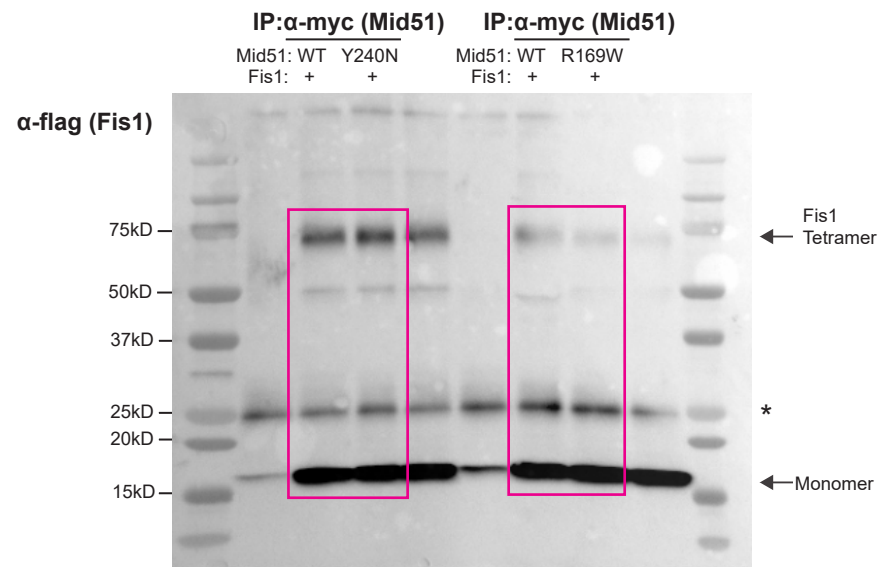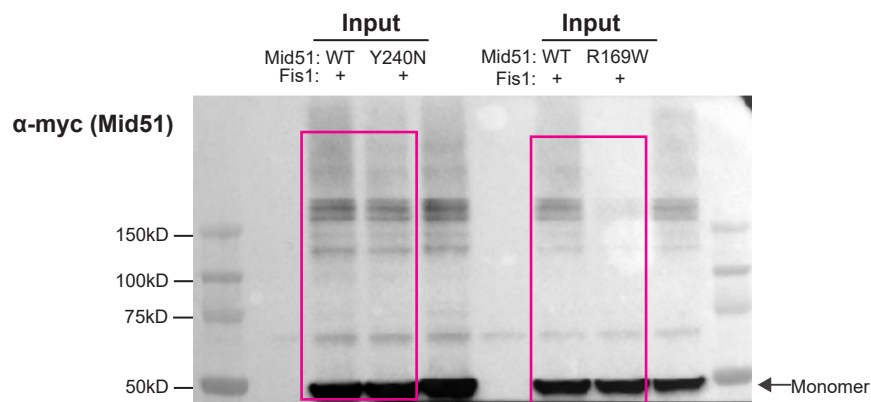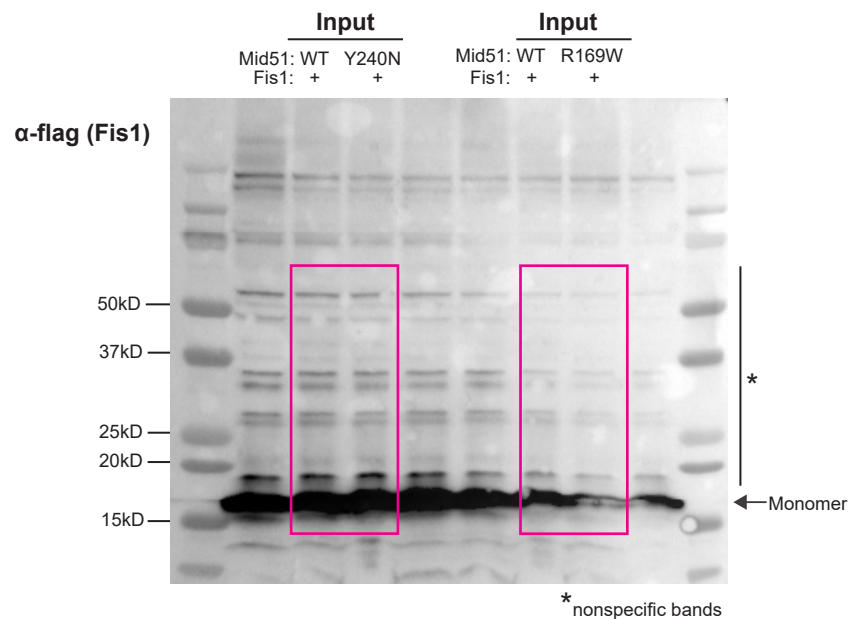

Supplement: SourceData F7 — is the source file for Fig. 7. [file JCB_202206140_SourceDataF7.pdf]

Figure S3A

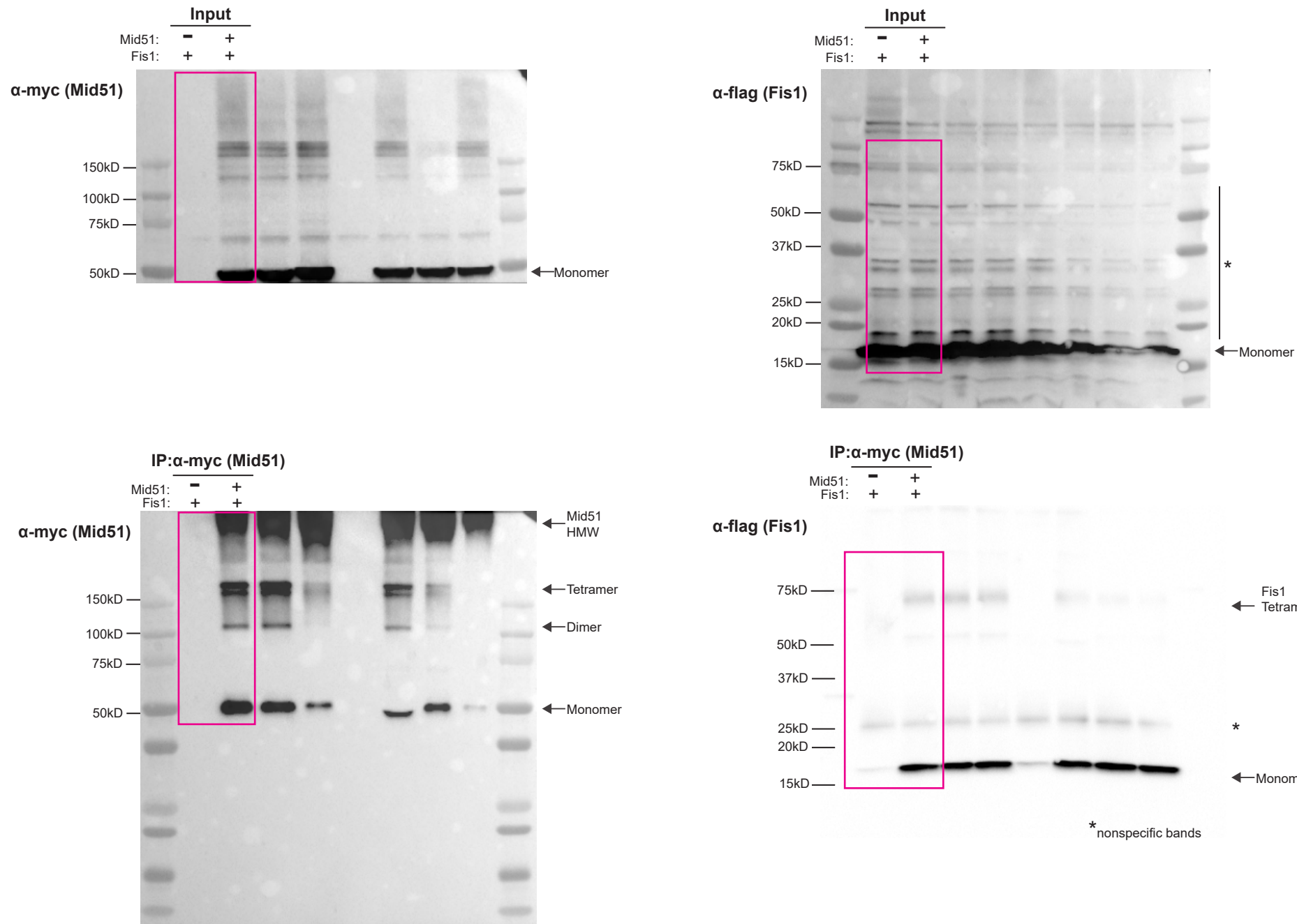

Figure S3B

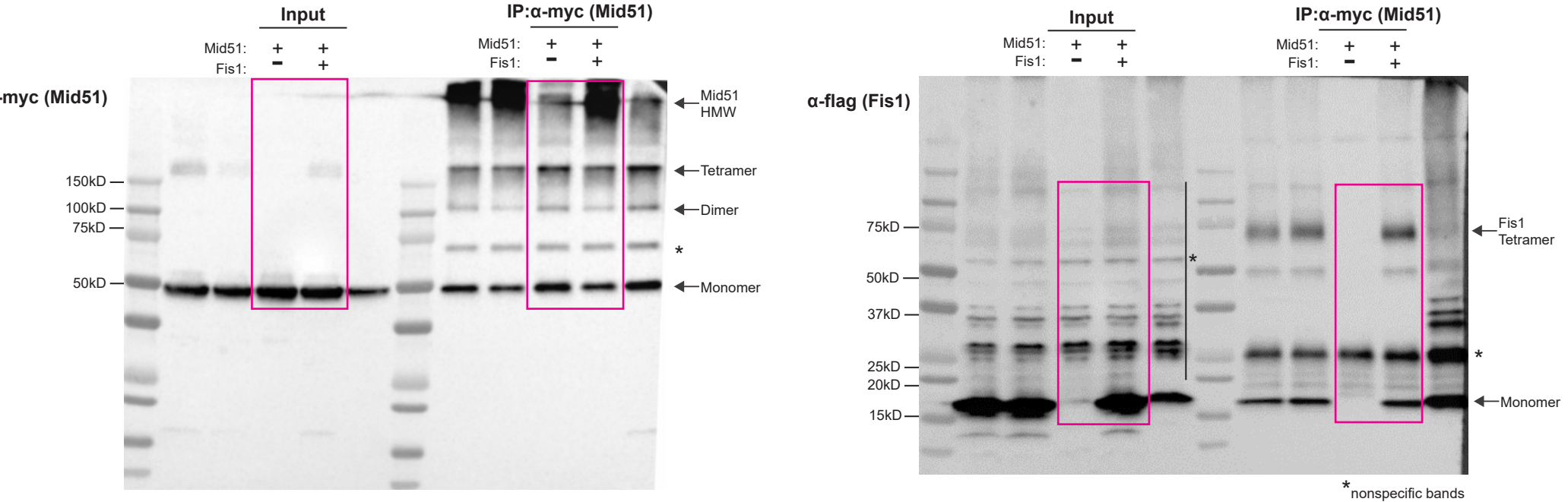

Supplement: SourceData FS3 — is the source file for Fig. S3. [file JCB_202206140_SourceDataFS3.pdf]
